# Anthracobunids from the Middle Eocene of India and Pakistan Are Stem Perissodactyls

**DOI:** 10.1371/journal.pone.0109232

**Published:** 2014-10-08

**Authors:** Lisa Noelle Cooper, Erik R. Seiffert, Mark Clementz, Sandra I. Madar, Sunil Bajpai, S. Taseer Hussain, J. G. M. Thewissen

**Affiliations:** 1 Department of Anatomy and Neurobiology, Northeast Ohio Medical University, Rootstown, Ohio, United States of America; 2 School of Biomedical Sciences, Kent State University, Kent, Ohio, United States of America; 3 Department of Anatomical Sciences, Stony Brook University, Stony Brook, New York, United States of America; 4 Department of Geology and Geophysics, University of Wyoming, Laramie, Wyoming, United States of America; 5 Department of Biology, Hiram University, Hiram, Ohio, United States of America; 6 Department of Earth Sciences, Indian Institute of Technology, Roorkee, Uttarakhand, India; 7 Department of Anatomy, College of Medicine, Howard University, Washington, District of Columbia, United States of America; Raymond M. Alf Museum of Paleontology, United States of America

## Abstract

Anthracobunidae is an Eocene family of large mammals from south Asia that is commonly considered to be part of the radiation that gave rise to elephants (proboscideans) and sea cows (sirenians). We describe a new collection of anthracobunid fossils from Middle Eocene rocks of Indo-Pakistan that more than doubles the number of known anthracobunid fossils and challenges their putative relationships, instead implying that they are stem perissodactyls. Cranial, dental, and postcranial elements allow a revision of species and the recognition of a new anthracobunid genus. Analyses of stable isotopes and long bone geometry together suggest that most anthracobunids fed on land, but spent a considerable amount of time near water. This new evidence expands our understanding of stem perissodactyl diversity and sheds new light on perissodactyl origins.

## Introduction

Anthracobunidae is a family of large mammals that is only known from the middle Eocene of south Asia. Historically, anthracobunids have played a prominent role in debates surrounding the origin of Tethytheria, the group that includes the living and extinct members of the placental mammalian orders Sirenia (sea cows) and Proboscidea (elephants). Anthracobunids are often considered to have branched off near the base of Tethytheria [1]–[8], sharing most recent common ancestry with either Proboscidea [1], [9]–[13], Sirenia [14], [15], or the extinct Desmostylia. However, until now, no cranial parts, only some partial dentitions and two postcranial elements (a scapula associated with a maxilla [11] and an isolated astragalus [13]), were known for the group.

The paucity of fossil evidence has left Anthracobunidae as a phylogenetically and adaptively enigmatic group [2], [8], [16]–[20], with little known about their diets, habitats, and time and place of origin. In light of this, anthracobunid genera have also been aligned with two other, non-tethytherian clades, including Artiodactyla [21]–[23] and Perissodactyla [24], as well as phenacodontids [25], [26], one of the extinct families of basal ungulates. The convergent evolution of “ungulate”-like features in Paenungulata (containing Tethytheria) and Laurasiatheria (containing Artiodactyla and Perissodactyla) revealed by molecular data [27] requires that Anthracobunidae be re-evaluated within this new phylogenetic context.

Here we describe new anthracobunid fossils from the Kuldana and Subathu Formations of the early-middle Eocene of Pakistan and India [28]–[32]. These new findings more than double the number of anthracobunid fossils, and include cranial and postcranial material. We test the phylogenetic relationships of anthracobunids using a taxonomically rich morphological data set and investigate anthracobunid diet and habitat. Our results have important implications for character transformations near the origins of Perissodactyla, the habitats of stem perissodactyls, and the early biogeography of Paenungulata and Perissodactyla.

## Methods and Materials

### Osteological Sample

All fossil specimen numbers and identifying information are listed in the Supplementary Information.

Fossils were collected in Punjab Province, Pakistan, under a collaborative agreement of Howard University and the Geological Survey of Pakistan (H-GSP). Localities where fossils were collected, with topographical coordinates, were described in Thewissen et al. [33]. Specimens were prepared in the U.S.A. at Northeast Ohio Medical University (NEOMED) and are catalogued using the H-GSP acronym and accessioned into the collections of the Geological Survey of Pakistan (GSP). Since the GSP is a branch of the Pakistani government, all collected fossils are the property of the Pakistani state. At present, fossils are for study in the U.S.A., but they will be returned to Pakistan and will be permanently housed at the Natural History Museum of Pakistan, located in Islamabad. GSP geologists are collaborators on all field work as part of their official assignment and all permitting is internal to the different Pakistani government agencies involved. All necessary permits were obtained for the described study, which complied with all relevant regulations.

We also studied fossils from the locality Kalakot in Jammu & Kashmir State, India, the location of which is described by Russell and Zhai [32]. This locality was discovered by A. Ranga Rao, a geologist of the Oil and Natural Gas Commission, and he quarried large quantities of fossiliferous rock matrix from this locality. After his death, we were given access to these by his widow, F. Obergfell. Fossils were prepared at NEOMED in the U.S.A., and are the property of the Rangarao-Obergfell Trust for Geosciences, located in Dehra Dun, India, and are catalogued using the acronym RR. They will be housed on the Trust campus on Rajpur Road in Dehra Dun.

### Nomenclatural Acts

The electronic edition of this article conforms to the requirements of the amended International Code of Zoological Nomenclature, and hence the new names contained herein are available under that Code from the electronic edition of this article. This published work and the nomenclatural acts it contains have been registered in ZooBank, the online registration system for the ICZN. The ZooBank LSIDs (Life Science Identifiers) can be resolved and the associated information viewed through any standard web browser by appending the LSID to the prefix “http://zoobank.org/”. The LSID for this publication is: urn: lsid:zoobank.org:pub:CD33F507-0D06-4DE1-A3C8-8BF9E7697114. The electronic edition of this work was published in a journal with an ISSN, and has been archived and is available from the following digital repositories: PubMed Central, LOCKSS.

### Phylogenetic Methods

The morphological character matrix is a modified version of that which was most recently employed by Barrow et al. [34], and includes 92 taxa and 403 morphological characters. Extant *Canis* and *Equus*, and the following extinct taxa, many of which are of uncertain supraordinal position, were added to the morphological character matrix of Barrow et al. [35] (with references provided for those taxa that were, in whole or in part, scored from the literature): *Radinskya yupingae* (?Perissodactyla; late Paleocene; China) [36]; *Paschatherium dolloi* (Louisinidae; late Paleocene-early Eocene; Belgium, France, England) [37]; *Phenacodus intermedius* (Phenacodontidae; late Paleocene-early Eocene; U.S.A.); *Cambaytherium* (Cambaytheriidae; early Eocene; India) [38]; *Gandheralophus minor* (Perissodactyla; early Eocene; Pakistan) [39]; *Gujaratia indica* (Artiodactyla; early Eocene; India); *Gujaratia pakistanensis* (Artiodactyla; early Eocene; Pakistan) [40]–[42]; *Homogalax protapirinus* (Perissodactyla; early Eocene; U.S.A.) [43]–[45]; *Meniscotherium chamense* (Meniscotheriidae; early Eocene; U.S.A.) [46]; *Protomoropus gabuniai* (Perissodactyla; early Eocene; Mongolia) [47]; *Teilhardimys* ( = *Microhyus*) *reisi* (Louisinidae; early Eocene; Portugal) [48]; *Heptodon* (Perissodactyla; early-middle Eocene; U.S.A.) [49]; *Hyrachyus modestus* (Perissodactyla; early-middle Eocene; U.S.A.); *Hallensia* (?Perissodactyla; middle Eocene; France, Germany) [50], [51]; *Namatherium blackcrowense* (Embrithopoda; middle? Eocene; Namibia) [52]; *Mesohippus* bairdi (Perissodactyla; late Eocene and early Oligocene; U.S.A.); *Behemotops katsuiei* (Desmostylia; early Oligocene; Japan) [53]; and *Paleoparadoxia* (Desmostylia; early-middle Miocene; Japan, U.S.A.) [54]–[57]; *Ocepeia daouiensis* (Ocepeiidae; middle Paleocene, Morocco) [58]; and *Nementchatherium rathbuni* (Macroscelidea; late Eocene; Libya) [59].

Whenever possible, characters were scored based on analysis of original fossil material and/or casts housed in either the Department of Vertebrate Paleontology at the American Museum of Natural History, the Department of Anatomical Sciences at Stony Brook University, or the Department of Anatomy and Neurobiology at Northeast Ohio Medical University. As noted above, some taxa and/or characters were scored from the literature. Some dental characters were rescored for *Arsinoitherium*, taking into account new evidence for cusp homologies provided by the more basal embrithopod *Namatherium* [52]. Furthermore, a new character state (“large, inflated”) was added for the character “M1-2 metaconules” (number 18532) to take into account the condition seen in anthracobunids and some other ingroup taxa, and the character “shape of astragalar ectal facet” was excluded, because it could not be consistently scored across the new taxa that were sampled.

The Laurasian “condylarths” *Meniscotherium*, *Paschatherium*, *Phenacodus*, and *Teilhardimys* were included because all have been identified as possible members of Afrotheria [18], [58], [60]–[64]. Phenacodontids have also played an important historical role in interpretations of perissodactyl origins [65]. The placement of these taxa in recent phylogenetic analyses has arguably been obscured by differences in taxon sampling or analytical methods; i.e., some analyses incorporated molecular data [61] while others did not [18], [62], [63], [66]; some analyses sampled numerous early Paleogene “condylarths” [18], [62], [63] while others sampled primarily extant taxa and only a few extinct species [61]. No consensus view has emerged from these studies. For instance, Thewissen and Domning found phenacodontids and meniscotheriids to fall outside of a paenungulate-desmostylian-perissodactyl clade [66]; Tabuce et al. [63] placed louisinids and anthracobunids as paenungulates, and phenacodontids as perissodactyls, while Zack et al. [62] placed louisinids with Macroscelidea [62]. The analyses of Gheerbrant et al. [58] placed phenacodontids as a sister group of a Macroscelidea-Perissodactyla-Paenungulata clade, and louisinids as either stem macroscelideans or stem members of a perissodactyl-paenungulate clade. Wible et al.'s [67] analysis placed Meniscotherium and Phenacodus close to early artiodactyls, far from paenungulates and macroscelideans. In Hooker and Russell's recent analysis of louisinid interrelationships [64], the group's macroscelidean affinities were assumed, not tested. Clearly, these enigmatic Laurasian “condylarth” taxa need to be analyzed within the context of a taxonomically broad morphological analysis that incorporates evidence from molecular data, thereby allowing for the independent evolution of “ungulate”-like features in Perissodactyla and in Afrotheria. The combined molecular-morphological analysis of Asher et al. [61] provided such a context, but their study did not sample a number of key fossil taxa, such as basal fossil hyracoids, proboscideans, sirenians, macroscelideans, and perissodactyls.

The matrix analyzed here included a total of 92 taxa and 403 characters (See Suppl. File). Rather than incorporate molecular data directly into the parsimony analysis, we employed molecular “scaffolds” based on the results of Meredith et al. [27] for placentals and Smit et al. [68] for relationships within Macroscelidea. For the primary analysis, the scaffold enforced the monophyly of the following clades: Afrosoricida (Chrysochloroidea + Tenrecoidea), Afroinsectivora (Macroscelidea + Afrosoricida), Afroinsectiphilia, Tubulidentata + Afroinsectivora), Paenungulata, Afrotheria, Euarchonta, Eulipotyphla, and Laurasiatheria. In addition, the Miocene aardvark *Myorycteropus* was constrained to group with extant *Orycteropus*; otherwise this taxon tended to form a clade with *Dasypus*, perhaps because both taxa have a full complement of peg-like featureless teeth, whereas these teeth have been reduced in *Orycteropus*. As in the original study of Seiffert [69], polymorphisms were scored as an intermediate character state rather than using standard polymorphic scoring (i.e., “0/1”). For analyses in which multistate characters with such intermediate polymorphic states were ordered, they were weighted either as 0.5 or 1.0; analyses were also run with all characters unordered and equally weighted. An additional perturbation that we employed to test for sensitivity of our results was the placement of Xenarthra to fit either the Atlantogenata (Xenarthra + Afrotheria) hypothesis or the Exafroplacentalia [Xenarthra + (Laurasiatheria + Euarchontoglires)] hypothesis. All analyses were run in PAUP 4.0b10 [70], with 10,000 random addition-sequence replicates. Bootstrap support was calculated in PAUP, based on 1000 pseudoreplicates.

### Bone Geometric Methods

Midshaft cross-sections of fossil and extant large-bodied ungulate long bones and ribs [71] were visualized via paleohistological sections or high resolution micro-CT scans. Bone compactness (total amount of bone per section) was calculated via published methods [72].

### Stable Isotope Methods

For the analysis of stable isotopes, three or more specimens of each species were analyzed (when available; see Tables S8 and S9) to provide an estimate of the population mean ± s.d. for carbon and oxygen isotope values [73] http://www.nature.com/nature/journal/v450/n7173/full/nature06343.html - B33. About 5 mg of enamel powder was collected from each specimen, either by drilling directly from the tooth or by grinding enamel chips in an agate mortar and pestle. Before collection, contaminants were removed by abrading the outer surface of the specimen after cleaning the outer surface to a depth of ∼0.5 mm using a DREMEL drill with a 1 mm diameter diamond-coated bit.

Preparation of powders for stable isotope analysis followed published methods [74], [75]. Powders were first transferred to 1.5 mL microcentrifuge vials and then soaked sequentially overnight in about 0.20 ml of a sodium hypochlorite solution (1–2 g dl-1) and then in about 0.20 ml of calcium acetate buffered acetic acid (pH about 5) following published procedures [31]. On addition of each reagent, samples were agitated for 1 min on a Vortex Genie vortex mixer. After each soak, the supernatant was removed by aspiration and the residual powder was rinsed five times with deionized water. Samples were then freeze-dried overnight and about 1.5 mg of powder from each was weighed into individual test tubes for analysis on a Thermo-Finnigan gas bench autosampler attached to a Thermo-Finnigan DeltaPlusXP continuous-flow isotope-ratio mass spectrometer at the University of Wyoming Stable Isotope Facility.

All values for stable isotopes are reported in delta (δ) notation, using the equation δ(‰)  = 1,000 × (Rsample/Rstandard - 1), where Rsample is the observed isotope ratio of the sample (^13^C/^12^C or ^18^O/^16^O) and Rstandard is the accepted ratio for an appropriate international standard (Vienna Pee Dee belemnite for δ^13^C; Vienna Standard Mean Ocean Water for δ^18^O). Analytical precision is typically better than 0.1‰ for δ^13^C values and 0.2‰ for δ^18^O values (±1σ).

Enamel δ^18^O values are influenced by the oxygen isotope compositions of oxygen sources (atmospheric O_2_, food and water ingested by the animal), certain physiological processes that affect intake or loss of oxygen by an animal (sweating, panting, respiration, and elimination of urine and feces), and body temperature [76]–[78]. For aquatic and semi-aquatic mammals, the flux of environmental water by means of direct ingestion and transcutaneous exchange [79] overwhelms all other oxygen sources, which reduces inter-individual variation [76], [80] and causes enamel δ^18^O values of freshwater taxa (for example Hippopotamus) to be lower than those for terrestrial mammals (∼2–3‰); [81]–[83]. The magnitude of offset between freshwater and terrestrial mammals can vary in response to changes in humidity, such that faunas experiencing drier conditions show a greater offset (∼3.0‰) than those living under wetter conditions (<1.0‰) [84].

In combination with enamel δ^18^O values, we also determined enamel δ^13^C values of fossil species, which were used to infer diet and past environmental conditions. For herbivorous ungulates, enamel carbon isotopic compositions are enriched in ^13^C relative to diet by ∼14‰ [85]. Variation in enamel δ^13^C values among ungulate species is most strongly affected by the carbon isotopic composition of the plants they ingest. In the Eocene, terrestrial ecosystems were dominated by plants performing C3 photosynthesis, a process that discriminates against the heavier carbon isotope (^13^C), causing plant tissue δ^13^C values to be considerably lower than those of plants exploiting other photosynthetic pathways (C4 plants, CAM plants) [86], [87]. The carbon isotopic compositions of C3 plants are more variable than other plant types and are strongly affected by temperature and aridity, causing C3 plants grown under hot dry conditions to have higher δ^13^C values than those grown in cooler and wetter environments [88], [89]. Since enamel δ^13^C values reflect the carbon isotopic composition of the plants in an animal's diet, consumer enamel δ^13^C values can be used to infer past environmental conditions (wet vs. dry) as well as dietary information for extinct species.

## Results

### Systematic Paleontology

Placentalia Owen, 1837; Order Perissodactyla Owen, 1848.

Family Anthracobunidae Wells and Gingerich, 1983.

*Type Genus. Anthracobune* Pilgrim, 1940.

*Included Genera. Jozaria* Wells and Gingerich, 1983; and *Obergfellia,* new genus.

*Diagnosis*. Stem perissodactyls with premolars increasing in complexity from anterior to posterior, but never molariform; molars brachyodont, but with high cusps and low valleys between cusps; upper molars increasing in size from M1 to M3; lower molars with a distinct trigonid elevated above the talonid; large caudally-projecting angular process of the mandible.

**Figure 1:**
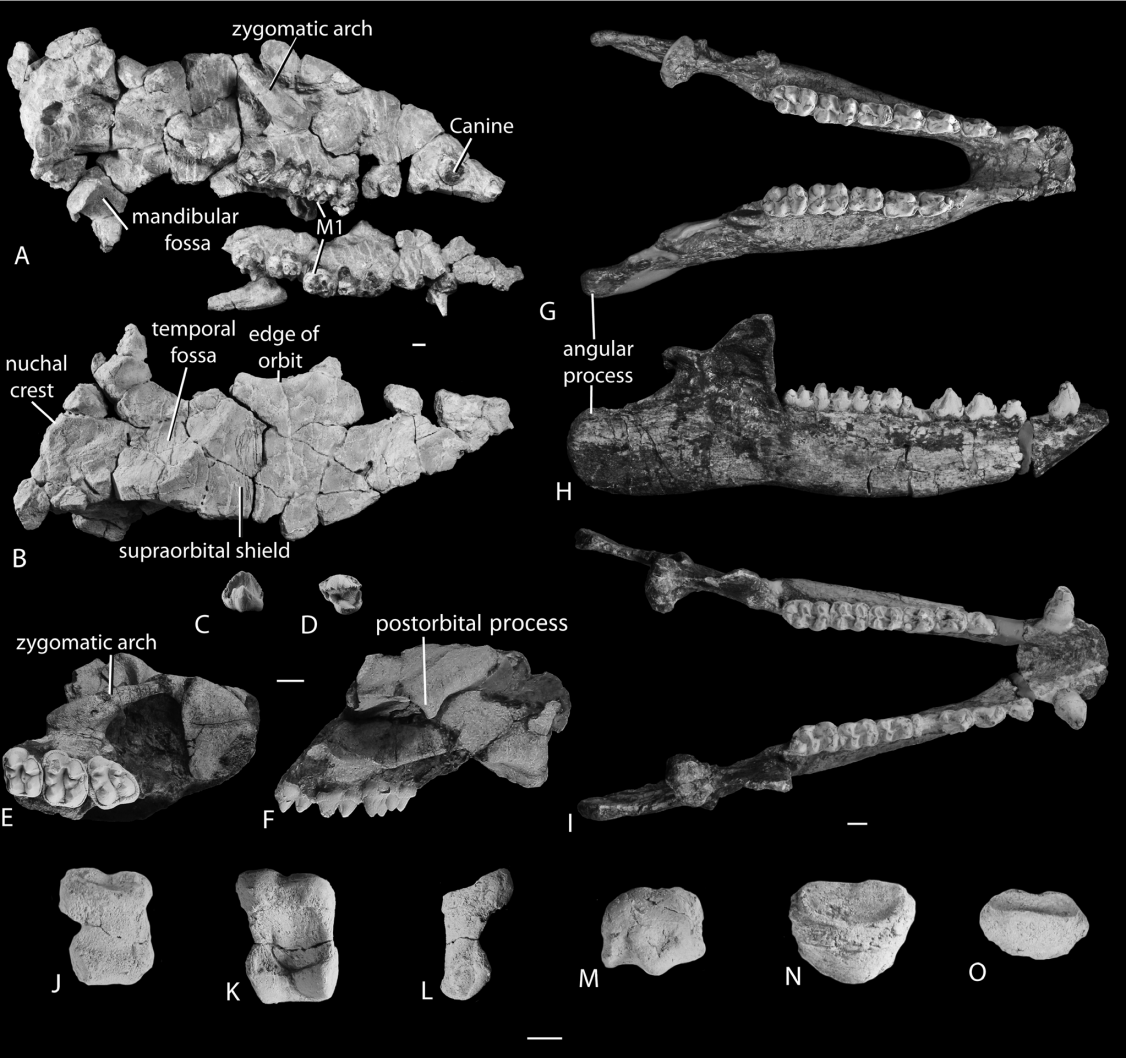
Cranial elements of anthracobunids from the middle Eocene of Indo-Pakistan. (*A*) Crushed skull of *A. pinfoldi* (H-GSP 97106) in ventro-lateral view (left maxilla detached) and (*B*) dorsal view; (*C*) P2 of *A. wardi* (H-GSP 30229) in lingual view and (*D*) occlusal view; (*E*) Skull fragment of *A. wardi* (RR 411) in occlusal view, and (*F*) lateral view; (*G*) Mandible of *A. wardi* (H-GSP 96434) in occlusal view; (*H*) Mandible of *A. wardi* (H-GSP 96258) in lateral view, and (*I*) occlusal view. (*J*) Proximal phalanx of *A. pinfoldi* (H-GSP 97106.105) in dorsal view; (*K*) Proximal phalanx of *A. pinfoldi* (H-GSP 97106.101) in ventral view, and (*L*) lateral view; (*M*) Head of a metapodial of *A. pinfoldi (*H-GSP 97106.250) in dorsal view; (*N*) Phalangeal fragment of *A. pinfoldi* (H-GSP 97106.257) in dorsal-superior view; (*O*) Terminal phalanx of *A. pinfoldi* (H-GSP 97106.302) in dorsal-superior view. Scale bar is 1 cm in length.

**Figure 2:**
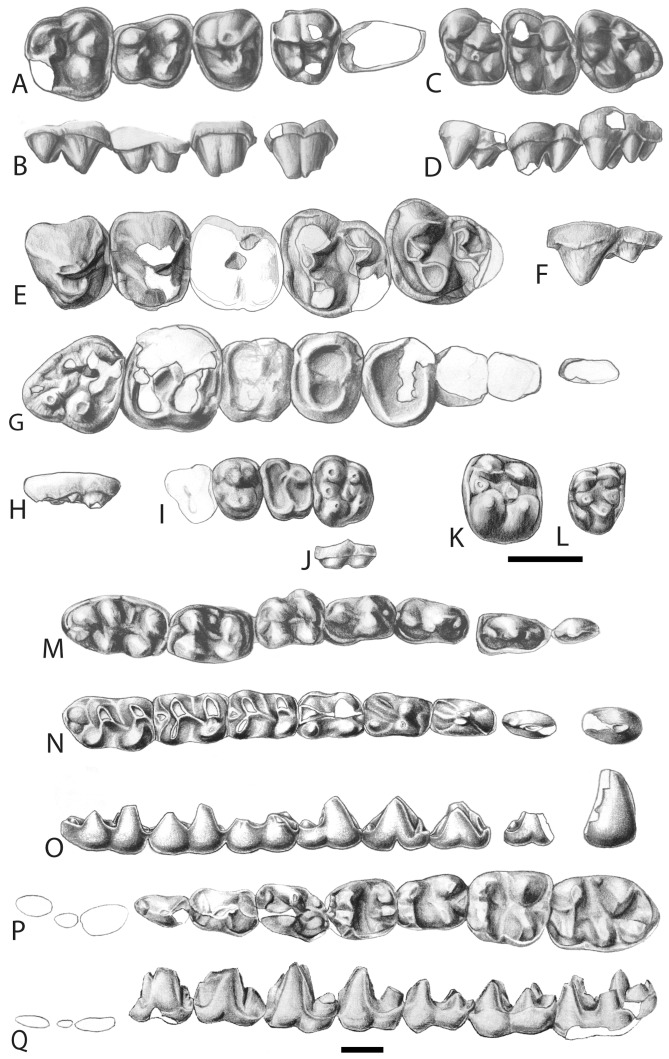
Teeth of anthracobunids, other stem perissodactyls, and condylarths. (*A*) Right P2-M2 of *A. wardi* (LUVP-15006) in occlusal view, and (*B*) labial view; (*C*) left M1-M3 of *A. wardi* (RR-411) in occlusal view, and (*D*) labial view; (*E*) left P3-M3 of *A. pinfoldi* (H-GSP 82-31P) in occlusal view; (*F*) left M3 of *A. pinfoldi (*H-GSP 82-31P) in labial view; (*G*) right C-M3 of the cambaythere *Kalitherium* (IITR-SB-VLM 931) in occlusal view; (*H*) right M3 of *Kalitherium* (IITR-SB-VLM 931) in labial view; (*I*) left P4-M2 of *Nakusia* [6] in occlusal view; (*J*) left M2 of *Nakusia* [6] in labial view; (*K*) Mx of *Cambaytherium* (IITR-SB-VLM-521) in occlusal view; (*L*) right M3 of the phenacodontid ‘condylarth’ *Tetraclaenodon* (KU-8052) in occlusal view; (*M*) left p1-m3 of *A. wardi* (WIF/A 1101) [15] in occlusal view; (*N*) left c-m3 of *A. wardi* (H-GSP 96258) in occlusal view, and (*O*) labial view; (*P*) left p1-m3 of *Obergfellia occidentalis* (H-GSP 1981) in occlusal view, and (*Q*) labial view (M1 inverted from right side). Scale bar is 1 cm in length. Illustrations by Jacqueline Dillard.

*Description*. Unlike tethytheres and other paenungulates, anthracobunids have small and simple upper and lower incisors and relatively large canines (Figs. 1, 2). Cheek teeth are bunodont and brachyodont and each cusp has a high apex, but there are deep valleys between cusps. Premolars are never totally molariform, and diastemata, if present at all, are short. Upper P3 and P4 are shorter than the molars, and have a paracone, protocone, and metacone, but no hypocone. The M1 and M2 have four well-developed cusps, as well as strong para- and metaconules, without lophs. The distance between proto- and paracone is longer than that between the hypo- and metacone, giving the tooth a posteriorly pinched appearance. Lower molars have a trigonid with two large cusps, and large, sometimes twinned, hypoconulid on the third lobe (Figs. 1G–I, S1).

Partial skulls are known for two anthracobunids (H-GSP 97106 and RR 411), and both show the presence of a very thick bony shelf over the orbits (Fig. 1B, F). The palate is concave mediolaterally, with the areas near the midline much higher than the alveolar processes. The lateral sides of the basisphenoid and basioccipital are recessed; this is particularly true for the basioccipital, which suggests that the petrosal, which is not preserved, was also deeply recessed. The mandibular fossa is concave and is located just lateral to the ear region. The postglenoid process is oval and does not extend along the entire width of the mandibular fossa.

H-GSP 97106 includes, among other elements, metapodials and phalanges (Fig. 1J-O). The heads of most metapodials are wider than deep, strongly convex, and the posterior side bears a strong crest. Proximal phalanges show a broad, oval articular surface for the metapodial and are squat: nearly as wide mediolaterally as they are long proximodistally. Their distal articular facet is rectangular in outline and deeply incised caudally, indicating the presence of a caudal crest on the middle phalanx. Most prominent on the proximal phalanges is the deeply excavated posterior side which is concave both mediolaterally and proximodistally.

*The genera of the family Anthracobunidae.* We restrict the family Anthracobunidae to the genera *Anthracobune* (which includes most specimens previously referred to *Pilgrimella* and *Lammidhania*), *Jozaria*, and the new genus *Obergfellia*. There are several other families of medium-sized bunodont “ungulates” from the Eocene of India and Pakistan, such as quettacyonids [90] and cambaytheres [4], [91]. While all of these groups are brachyodont, anthracobunids are unique among them in having tall cusps on their molars, while retaining valleys between the cusps that are low and close to the cingulum (Fig. 2). The new genus *Obergfellia* differs from other anthracobunids in exhibiting the following combination of features: (*i*) lower molars broad, (*ii*) lower m3 relatively short, and (*iii*) angular process of the mandible long but shorter than that of *Anthracobune* (Figs. 1, 2, S1.). The last of these features is not known in *Jozaria*.

We exclude *Ishatherium subathuensis* from anthracobunids. Its holotype consists of the lingual side of an upper molar, referred originally to Sirenia [15], [92]. Wells and Gingerich [1] referred *Ishatherium* to Anthracobunidae. The holotype and only specimen shares with anthracobunids the strong development of conules and the deep transverse wear along the protocone-paraconule-paracone and along the hypocone-metaconule-metacone. The specimen is decidedly unlike anthracobunids in that the distance between protocone and paracone is similar to that between hypocone and metacone, and in that its cusps are not highly raised. We also exclude *Nakusia shahrigensis* [6] from anthracobunids (Fig. 2 I,J). Its holotype is a maxilla with P4-M2 and the base of P3. The base of P3 is elongate, the cusps on the relatively unworn M2 are low, and the molars are relatively wide. Anthracobunids have a short P3, high molar cusps, and squarish upper molars. *Nakusia* is more likely a quettacyonid or cambaythere than an anthracobunid.

The genus *Indobune* was included in Anthracobunidae [4], but it has low cusps on its teeth and is better classified as a cambaythere. Ducrocq et al. [7] described *Hsanotherium parvum* on the basis of two specimens with upper molars from Myanmar. These specimens are bunodont, with small conules and lack a hypocone. The transverse wear pattern of anthracobunids is also absent. *Hsanotherium* displays similarities to medium-sized bunodont artiodactyls, such as *Haqueina* [22], and we do not include it in anthracobunids.

*Anthracobune* Pilgrim, 1940.

*Type Species. Anthracobune pinfoldi* Pilgrim, 1940.

*Referred species. Anthracobune wardi* (Dehm and Oettingen-Spielberg, 1958).

*Diagnosis*. Anthracobunid with narrow lower molars and a very large angular process of the mandible (Fig. 1, Fig. S1).

*Discussion.* We refer to most of the specimens formerly included in *Lammidhania* and *Pilgrimella* in the past to *Anthracobune.*

*Anthracobune pinfoldi* Pilgrim, 1940.

*Holotype*. BMNH M.15792, left m2–3 and right m3, from ‘*Lammidhan*’ and ‘*Planorbis* freshwater beds.’ Pilgrim never visited the type region, and the fossils were collected by a geological surveyor (T. G. B. Davies). *Lammidhan* appears on Davies’ unpublished map and is located on a broad alluvial plain. However, it does not match a modern topographical landmark and its meaning is not known to local people. Eocene deposits occur to the north and south of the plain, with marine limestones and muds forming ledges usually towering over the unconsolidated muds that form the bottoms of valleys. These muds are the “*Planorbis* Freshwater beds,” and it is our contention that the specimen may have been found on these beds, but that it rolled down from overlying marine beds. Gingerich [23] improved the holotype by fitting BMNH M.15794 (the trigonid of the left M2) to it.

*Diagnosis*. Large species of *Anthracobune*.

*Referred Specimens.* See Suppl. Info.

*Description*. H-GSP 97106 is the most complete specimen known for any anthracobunid. Upper incisors are similar in size (based on their alveoli); the crown of I3 shows that there is a single, large pointed cusp, with small basal thickening on the cingulum anterior and posterior to it. Canine is long and compressed mediolaterally and P1 is two-rooted, with single cusp. P2 has a small protocone and two cuspules on the posterior crest of the paracone. P3 has a protocone, paracone, metacone and two conules, and a strong postprotocrista. Upper molars with four main cusps as well as strong para- and metaconule. Instead, protocone-paraconule-paracone have aligned wear surfaces, and similar wear surfaces on hypocone-metaconule-metacone. With wear, these surfaces resemble crests.

Lower p1 with two roots, strong protoconid, high but narrow paraconid and low metaconid, lacking talonid. Lower p2 is similar to p1 but larger. Lower p3 and p4 are similar with a well-developed trigonid having three cusps, and two cusps on the talonid. Lower molars with strong crests between protoconid and metaconid, and between hypoconid and entoconid; weak hypoconulid on anterior molars. These crests are stronger than the cristid obliqua. Paraconid usually absent on molars.

Upper anterior teeth lined up in a parasagittal row, and rostrum ends in a narrow point. The caudal edge of the nasal opening of H-GSP 97106 is over the upper canine, and the infraorbital foramen is dorsal to P3. The zygomatic arch projects lateral from the base of M1 and M2, and extends caudally from there. The orbit is located over M2 and M3. The skull roof dorsal to the orbits is a thick bony plate that is flat and stretches rostrally to the nasal aperture, and caudally to the temporal crests. Left and right temporal crests form the caudal extent of this plate and join into a strong sagittal crest that delineates the large temporal fossae. This crest ends at the left and right nuchal crests, which extend ventral to the ear region.

The palate is narrow rostrally, and concave mediolaterally. A weak postpalatine torus is present, and the hard palate is indented caudally with the choana reaching rostrally medial to M3. The pterygoid process is thick. The periotics are deeply recessed between basioccipital and mandibular fossa. Basisphenoid and basioccipital slope strongly from medial to lateral. The postorbital process is strong and oval in cross-section, the mandibular fossa is cylindrical, its rostrocaudal extent is as large as its mediolateral extent. The external auditory meatus is immediately caudal to the postorbital process, there is no postglenoid foramen. The external auditory meatus is directed caudolaterally. The suture between mastoid and squamosal is on the nuchal crest, and there is a mastoid emissary foramen just below it. A large emissary foramen also occurs on the parietal in the temporal fossa. The foramen magnum faces more caudal than ventral and the supraoccipital immediately dorsal to it is flat. Near the sagittal crest, the supraoccipital plane is concave.

*Discussion.* Specimens referred to *A. pinfoldi* are known from the upper part of the Kuldana Formation. These are coastal beds, as indicated by the sedimentology and invertebrate paleontology of their localities, although isotopic evidence implies that the individuals fed on land. The upper part of the Kuldana Formation is well known for its fossil whales: *Ambulocetus* and *Attockicetus*.

*Anthracobune wardi* Dehm and Oettingen-Spielberg, 1958.

*Holotype*. BMNH M.15799, isolated talonid, most likely of m1 based on its size. Locality given as ‘*Lammidhan*…*Planorbis* freshwater beds of Chharat stage’ (see discussion under holotype of *Anthracobune pinfoldi*). Unlike other specimens from Pilgrim's ‘*Lammidhan*,’ this specimen is probably indeed from the *Planorbis* beds, an inference based on its preservation.

*Diagnosis*. Lower molars with high length/width ratio, the m3 short, paraconid and metaconid of lower premolars small, anterior premolars long.

*Referred Specimens.* See Suppl. Info.

*Description*. New material referred to this species includes two complete lower jaws that include the entire post-incisor dentition (H-GSP 96258 and 96434), a skull fragment that includes orbit and supraorbital region (RR 411), and part of the deciduous dentition.

Lower incisors are similar in size to each other, based on their alveoli. Canines with short crown, larger than the incisors, small diastemata on either side of p1, but no other diastemata present. Lower p1 and p2 with one main cusp and two roots, and small cusp on pre- and postprotocristid. Lower p3 and p4 with well-developed trigonid and talonid. Protoconid and metaconid on these teeth similar in height and paraconid lower, much lower on p4. Talonid of posterior premolars with high hypoconid, and entoconid high or barely distinguishable. Lower molars with distinct trigonid and talonid. Protoconid and metaconid similar in height, paraconid absent, but paracristid well developed. Hypoconid and entoconid similar in height, cristid obliqua strong, hypoconulid weak on m1 and m2, but strong and placed on third lobe in m3, and sometimes twinned. Coronoid process of the mandible small and with steep anterior slope. Length of mandible anterior to anterior edge of coronoid process (to rostral end of the fused symphysis) is approximately two times as long as that posterior from this edge (to the tip of the angular process). This indicates that the angular process is enormous. Lower p3 with three cusps on trigonid, and two on talonid; trigonid long. Lower p4 with shorted trigonid, resembling lower m1 (H-GSP 96052). Mandibular angle of juveniles small (H-GSP 30349, Fig. S1).

Upper dentitions of *A. wardi* are mostly known from India (LUVP 15006, RR 411, WIF/A 616). The P2 is longer than wide, unlike the P3 and P4. P2 has connate paracone and metacone, and a lingual bulge with a small protocone (LUVP 15006, H-GSP 30229). P3 and P4 are similar in shape, with connate para- and metacone, and large protocone. Paraconule and metaconule are strong in P3 (LUVP 15006), whereas in P4 transverse crests are well developed and conules less so. Upper molars have four strongly developed cusps, with smaller but distinct paraconule and metaconule. Transverse crests are absent, but cusps are lined up in such a way that they form an anteriorly convex arch.

Tooth wear in anthracobunids tends to be distinct on the posterior side of the protoconid-metaconid, and the anterior side of the protocone-protoconule-paracone, and is accentuated by the deep valleys behind these rows. This transverse wear pattern is consistent with the large and caudally-projecting angular process of the mandible, the area of insertion for the medial pterygoid and masseter muscles.

Specimen RR 411 reveals some details about the orbit and zygomatic arch. The zygomatic arch has a weak postorbital process and the dorsal part of the zygomatic arch is made up of the maxilla in the orbital rim, but not posterior to the orbit. The ventral edge of the zygomatic arch is made up of the jugal. The morphology of the root of the zygomatic arch, the postorbital process of the frontal, and the mandibular fossa in this specimen resembles that of H-GSP 97106.

The anterior edge of the ascending ramus of the mandible of H-GSP 96259 is vertical, whereas it slopes slightly caudal in H-GSP 96434: the superior part overhanging the inferior part. H-GSP 30349 is a juvenile in which the anterior edge of the ramus slopes slightly rostral. In H-GSP 96149 this ramus is vertical. The mandibular symphysis of all jaws, except H-GSP 30349, is strongly fused, and slopes caudal ending ventral to the premolars.

*Discussion*. Most specimens of *Anthracobune wardi* are known from the lower redbeds of the Kuldana Formation of Pakistan, where pakicetid cetaceans are common. These are freshwater deposits and the specimens of *Anthracobune* found here pertain to *A. wardi*. Additional specimens of *A. wardi* are from the freshwater deposits of the Subathu Formation of India.

*Obergfellia*, new genus. urn:lsid:zoobank.org:act: 952704F2-F029-4643-B791-B30E7C71AA0A.

*Anthracobune* (in part), West, 1980, 1981, 1983, 1984.

*Pilgrimella* (in part), Wells and Gingerich, 1983; Kumar, 1991.

*Type and only species. Obergfellia occidentalis,* new species.

*Etymology.* In honor of the late vertebrate paleontologists Dr. Friedlinde Obergfell and her husband A. Ranga Rao. Both were instrumental in the initial discovery and description of the Kalakot fauna. This fauna became the best-known Eocene land mammal fauna from India, mostly due to the efforts of A. Sahni and K. Kumar.

*Distribution.* Middle Eocene, northern India and Pakistan.

*Diagnosis*. As for the type species until other species are described.

*Obergfellia occidentalis*, new species, urn:lsid:zoobank.org:act:B5A9A6DA-DB8F-4498-8BF2-3812B98458D9.

*Anthracobune pinfoldi* (in part), West, 1980, p. 518, Pl. 2.5.

*Anthracobune pilgrimi*, West, 1981; West, 1983 (in part), p. 367, [Fig pone-0109232-g003] [Fig pone-0109232-g004] Fig. 5, Fig. 6; West, 1984 (in part), p. 187, Fig. 4, Fig. 5.

**Figure 3 pone-0109232-g003:**
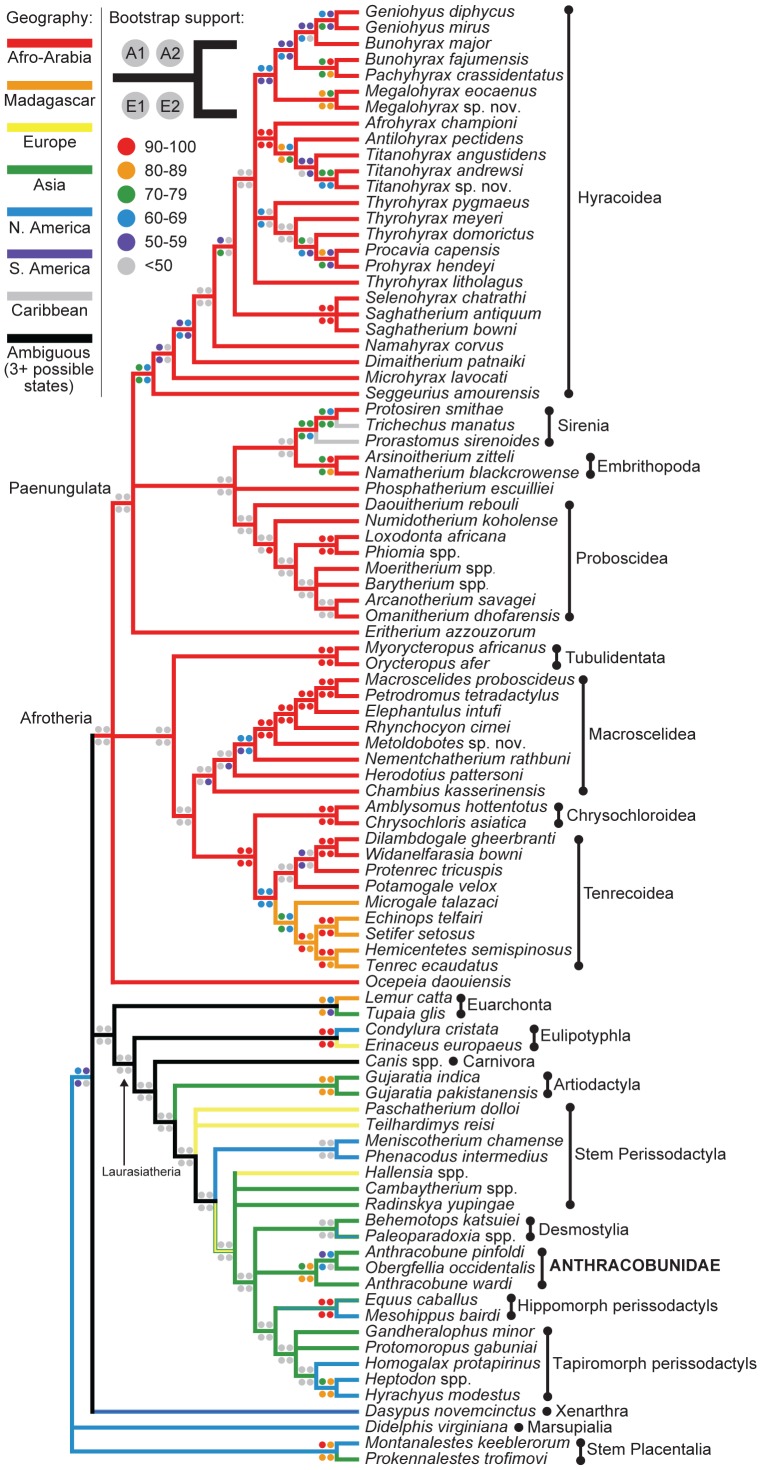
Adams consensus of all trees recovered from parsimony analyses that included some ordered multistate characters, with continental geography (see states in upper left-hand corner) optimized onto the tree using parsimony (relationships among extant taxa were constrained by a “molecular scaffold”). Bootstrap support for clades derived from each analysis contributing to the consensus is depicted by colored circles. A1  =  Atlantogenata constraint, transitions between polymorphic and “fixed” states in ordered morphoclines weighted as 0.5 steps; A2  =  Atlantogenata constraint, transitions between polymorphic and “fixed” states in ordered morphoclines weighted as one step; E1  =  Exafroplacentalia constraint, transitions between polymorphic and “fixed” states in ordered morphoclines weighted as 0.5 steps; E2  =  Exafroplacentalia constraint, transitions between polymorphic and “fixed” states in ordered morphoclines weighted as one step. Across all trees, anthracobunids and desmostylians were placed as perissodactyls, along with two enigmatic Asian taxa, the late Paleocene “condylarth” *Radinskya* and early Eocene *Cambaytherium*.

**Figure 4 pone-0109232-g004:**
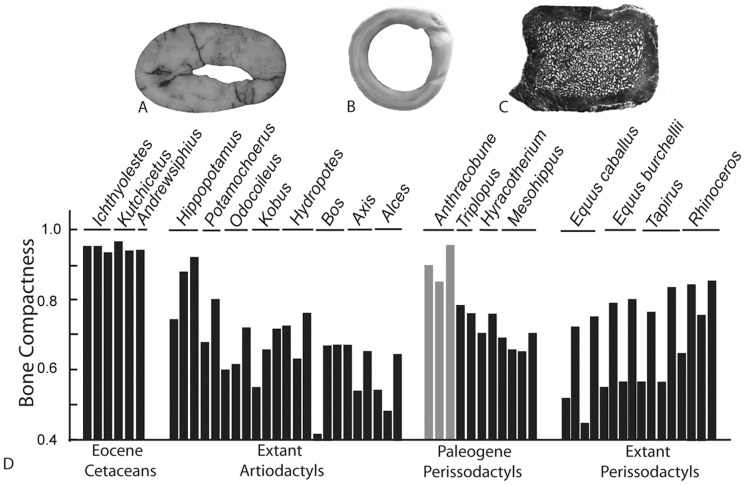
Histological midshaft sections for the (*A*) humerus of *Ichthyolestes* (H-GSP 96227), (*B*) femur of *Odocoileus*, and (C) radius of *Anthracobune* (H-GSP 97106). (*D*) Bar diagram of bone compactness, a quantification of the amount of bone per midshaft cross-section, compared here between fossil and extant ungulates.

**Figure 5:**
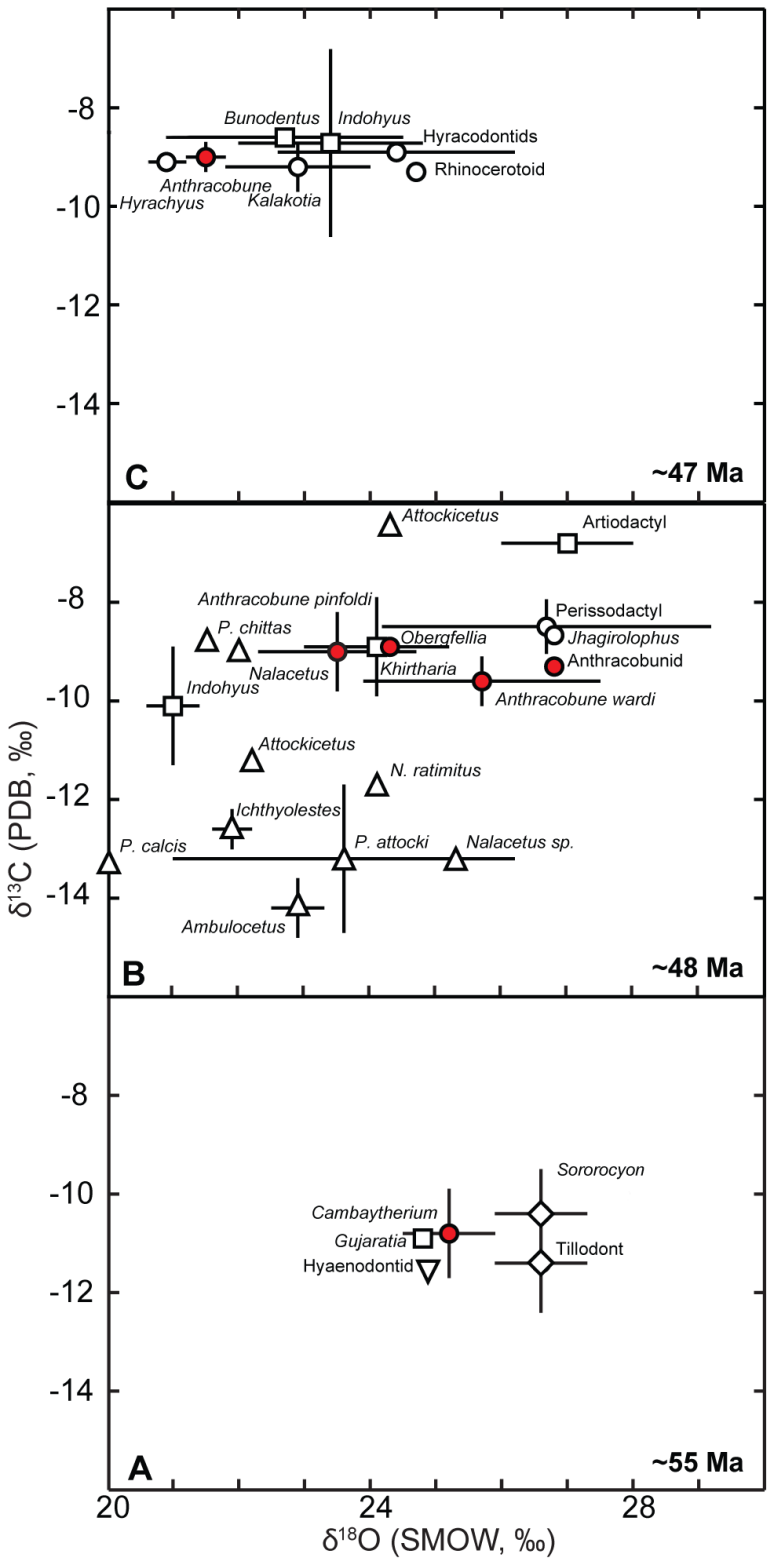
Bivariate plot of δ^18^O and δ^13^C values for enamel samples of early and middle Eocene mammals from India and Pakistan. Results shown as mean ±S.D. for the sample populations. Data from (*A*) early Eocene and (*B, C*) middle Eocene taxa from India and Pakistan. Circles, perissodactyls; red circles, anthracobunids; squares, artiodactyls; triangles, cetaceans; inverse triangles, creodonts; diamonds, condylarths. See Suppl. Info. for details.

*Pilgrimella pilgrimi,* Wells and Gingerich, 1983 (in part), p. 122, Fig. 2C–D; Kumar, 1991 (in part), p. 230.

*Etymology.* Specific epithet is from *occidentalis*, Latin for west, in honor of Robert M. West, who discovered and described the holotype.

*Holotype.* H-GSP 1981, mandibles with left p1-m3 and right p4-m3 (Fig. 2P–Q).

*Type Locality*. H-GSP Locality 62, Ganda Kas Area, Punjab Province, Pakistan.

*Formation and Age*. Kuldana Formation, Kala Chitta Hills of Northeastern Pakistan, early Lutetian in age (≈48 Ma).

*Diagnosis*. Differs from other anthracobunids in exhibiting the following combination of features: (*i*) lower molars broad, (*ii*) lower m3 short, and (*iii*) angular process of the mandible long but shorter than that of *Anthracobune*.

*Referred Specimens.* See Suppl. Info.

*Description*. The most complete specimen for this species is H-GSP 1981, a left and right mandible not larger than *A. wardi* (H-GSP 96258 and 96434), but with lower molars much broader than *A. wardi*. H-GSP 1981 lacks the angular process, due to post-mortem damage but it is preserved in H-GSP 96214, a specimen with an erupting m3. In this specimen, the angular process is 80% as long, rostro-caudally, as in *A. wardi*. The angular process is not preserved in H-GSP 96149, but its root is preserved and the slope thereof indicates that the process is small H-GSP 96149 has extremely worn teeth, but its molars are similar in size to H-GSP 1981 (Fig. S1), premolars and canine are larger and the depth and robusticity of the jaw is much greater than in H-GSP 1981. The wear stage is most consistent with advanced age, and makes it unlikely that the smaller angular process is the result of young age. Eventually this specimen may be shown to pertain to a new species of *Obergfellia*.

Few upper molars have been discovered for *O. occidentalis*. H-GSP 538 is a large tooth, and an interstitial facet on its posterior side indicates that it is not an M3. M1 and M2 of *P. pilgrimi* do not reach this size, and we therefore refer this tooth to *P. obergfelli*.

*Discussion.* West [10], [93] recognized that his collection of specimens from the Ganda Kas Region of Pakistan contained two species of anthracobunids from the freshwater part of the formation and used the names *Anthracobune pilgrimi and Lammidhania wardi* for them. Unfortunately, the type specimen for *L. wardi* is an isolated trigonid, and pertains to the same species as several large complete lower jaws collected by us (H-GSP 96258 and 96434), and these match upper dentition material referred to *Pilgrimella pilgrimi* (the type of which is an upper molar). Hence a new genus and species name is needed. A specimen described by West, H-GSP 1981, is the most appropriate holotype for this species.

*Jozaria* Wells and Gingerich, 1983.

*Jozaria palustris* Wells and Gingerich, 1983.

*Discussion*. Wells and Gingerich [1] described *Jozaria palustris* on the basis of a single specimen which shows the diagnostic features of anthracobunids. The lower premolars of this specimen are large compared to the molars, confirming it as distinct from other anthracobunid species and genera.

### Phylogenetic Results

Parsimony analyses were first run to determine the most parsimonious placement of Xenarthra (represented in this analysis by *Dasypus*) given different placements in placental phylogeny – i.e., as either the sister group either of Afrotheria (the Atlantogenata hypothesis) or of Laurasiatheria + Euarchontoglires (the Exafroplacentalia hypothesis), as this is one of the nodes that was not convincingly resolved by Meredith et al.'s [27] analysis, and continues to be debated in the literature on placental phylogenetics [94]. A placement with Afrotheria was more parsimonious [(3704.5 versus 3716 steps (i.e., constraint A1 versus E1, with 0.5 weighting of ordered multistate characters with intermediate polymorphic states; Fig. S2A versus Fig. S2C) and 5758 versus 5773 steps (A2 versus E2, no weighting of ordered multistate characters with intermediate polymorphic states, Fig. S2B versus Fig. S2D)], and all subsequent alternative constraints accordingly employed an Atlantogenata, rather than an Exafroplacentalia, constraint.

Regardless of whether Atlantogenata or Exafroplacentalia was constrained (assumption sets A1 and A2, and E1 and E2 respectively) anthracobunids and desmostylians were placed as derived stem perissodactyls (Fig. S2). In A1 and E1, desmostylians were joined by *Cambaytherium* and *Radinskya* as consecutive sister taxa (Fig. S2A and S2C). Anthracobunidae was always monophyletic in A1, A2, E1, and E2, and given the Adams consensus of all trees recovered by these analyses (Fig. 3), 16 character changes emerge as unambiguous synapomorphies of the family (Table S1, e.g., an enlarged p3 paraconid, deep hypoflexids on m2, the presence of two or more cusps on the m3 hypoconulid lobe, and reduction of the P3-4 metacones and metaconules). A placement of Desmostylia outside of Tethytheria and Paenungulata, and within Laurasiatheria, is not unprecedented: in a small number of Asher et al.'s [61] analyses, the desmostylian *Paleoparadoxia* was placed along the stem lineage leading to Perissodactyla and Cetartiodactyla (see their Table 6). Placements of non-perissodactyl and non-paenungulate fossil taxa were largely stable across all of these analyses, but relationships of stem and crown perissodactyls were not. Bootstrap support along the perissodactyl stem lineage was consistently <50%, but it should be noted that support was also <50% for several clades (e.g., Afroinsectivora, Afrotheria, Paenungulata, Laurasiatheria) that are strongly supported by maximum likelihood and Bayesian analyses of molecular data [27], [95]–[98]. Low support values for several nodes are probably due to dense sampling of incomplete fossil taxa that might decrease stability, but preserve intermediate states that break up long branches [99], [100]. Within Afrotheria, embrithopods were always placed as a sister group of Sirenia, while *Eritherium*, which has recently been described as a basal proboscidean, never grouped with Proboscidea but rather was placed either as a stem paenungulate (A1 and A2) or as a stem hyracoid (E1 and E2). The younger and more derived *Phosphatherium* was placed as a stem proboscidean in A1 and E1, but as a stem tethythere in A2 and E2. The recently described *Ocepeia* [58] was placed as either a basal atlantogenatan (A1), a stem atlantogenatan (A2), or as a stem paenungulate (E1 and E2).

When anthracobunids were constrained to join Paenungulata and certain multistate characters were ordered (Fig. S3A, Fig. S4A), the group joined Sirenia (along with Desmostylia and *Cambaytherium*); when all characters were unordered, anthracobunids were placed with Hyracoidea (Fig. S5). None of these alternative trees were rejected by non-parametric Templeton tests (Table S4, Table S5, Table S6). Placement with Sirenia required 6.5 steps more than the most parsimonious trees from A1, and 14 steps more than the most parsimonious trees from A2, while the placement with Hyracoidea only required an additional two steps with all characters unordered. Louisinids and phenacodontids remained as stem perissodactyls despite the constraint. When Desmostylia was constrained to group with Paenungulata, the clade again joined Sirenia, requiring an additional 3.5 steps over the most parsimonious trees from A1 (Fig. S3B), and eight steps over the most parsimonious trees from A2 (Fig. S4B). Given this constraint, anthracobunids did not join this desmostylian-sirenian clade, and remained as stem perissodactyls. Constraining *Eritherium* and *Phosphatherium* to be stem proboscideans (Fig. S3C, Fig. S4C, Fig. S7B) did not have an effect on the placement of either Anthracobunidae or Desmostylia, both of which formed a clade with Perissodactyla regardless of how characters were weighted. Finally, forcing *Cambaytherium* to join Anthracobunidae (Figs. S3D and S4D) to the exclusion of hippomorphs and tapiromorphs also did not affect the placement of these taxa as perissodactyls.

The Adams consensus of all trees recovered across A1, A2, E1, and E2 (Fig. 3) reveals that two enigmatic Asian taxa, late Paleocene *Radinskya* [36], and early Eocene *Cambaytherium*, are consistently situated close to Anthracobunidae as stem perissodactyls. *Radinskya* has long been an enigmatic figure in the study of perissodactyl origins. The genus was originally identified as a possible phenacolophid, albeit a “perissodactyl-like” one, by McKenna et al. [36], and was later placed outside of a perissodactyl-paenungulate clade in the phylogenetic analyses of Fischer and Tassy [17]. Both of these studies were undertaken prior to the publication of molecular sequence data that now strongly suggest that “Ungulata” is di- or polyphyletic, and that Paenungulata and Perissodactyla evolved their morphological similarities convergently [101]; *Radinskya* has not subsequently been included in combined analyses of molecular and morphological data. In our analyses *Radinskya* was always placed as a perissodactyl, but not in a consistent position relative to other stem and crown perissodactyls – either as a sister group of desmostylians and *Cambaytherium* (A1, E1, and E2), or in an unresolved position along the stem lineage of Perissodactyla (A2). The analyses of unordered and unweighted characters recovered hundreds of equally parsimonious trees, the strict consensus of which was poorly resolved, but *Radinskya* and *Cambaytherium* occasionally joined Anthracobunidae in some trees.

The placement of *Cambaytherium* within Perissodactyla is consistent with Bajpai *et al.*'s [38], [91] assessment of the genus. Confusion surrounding the dental similarities that *Cambaytherium* shares with perissodactyls *and* anthracobunids [102] is resolved by the placement of cambaytheriids and anthracobunids as closely related sister taxa of crown Perissodactyla, thereby indicating that similarities to tethytheres are entirely due to convergence. Bajpai *et al*. [38] argued that the middle Eocene European genus *Hallensia*, which was originally identified as a “condylarth” [50] and later placed within Perissodactyla as either a possible equoid [51], [103], might be a cambaytheriid. In our analyses *Hallensia* was usually placed as a stem perissodactyl (A1, E1, E2) or in an unresolved position relative to these taxa (A2). The monophyly of a clade containing Anthracobunidae, *Cambaytherium*, *Hallensia*, *Radinskya*, and “advanced” perissodactyls is supported by 11 unambiguous synapomorphies (Table S2). The clade containing anthracobunids, cambaytheriids, *Radinskya*, crown Perissodactyla, and *Hallensia* was reconstructed as having been equivocally of either Asian or European origin (Table S3), while the anthracobunid-desmostylian-hippomorph-tapiromorph clade was optimized as having been of Asian origin.

### Bone Geometry Results

We quantified the midshaft cross-sectional geometries of limb and rib bones of anthracobunids and other ungulates. Long bone and rib cross-sectional phenotypes are a reliable indicator of vertebrate habitat, with most taxa occupying a shallow-water habitat displaying the greatest amount of bone per cross-section via thickened cortices and/or bone-filled medullary cavities [71], [104], [105]. In contrast, bones of terrestrial taxa typically display thin cortices and vacant medullary cavities. Bone compactness values, a combined measure of cortical and medullary bone area [72], indicate that *Anthracobune* limb bones display values between 0.85–0.96. This range is greater than values observed in a sample of fossil and extant artiodactyls and perissodactyls (0.42–0.83), with the exception of modern *Hippopotamus* (0.94, 0.96) and *Rhinoceros* (0.64–0.85), and semi-aquatic pakicetid and remingtonocetid Eocene cetaceans (0.93–0.96) (Fig.4). Similar results were documented in rib elements (Table S7).

### Stable Isotope Results

We also studied the oxygen and carbon isotope composition of structural carbonate in tooth enamel [75], [106] for anthracobunids and coeval taxa from the Early and Middle Eocene of India and Pakistan (Fig. 5, Table S8, Table S9). Mammal fossils sampled from Early Eocene localities (Fig. 5C), including *Cambaytherium*, spanned a narrow range of δ^18^O values, and are consistent with occupation of a wet and forested habitat. Enamel δ^13^C values showed more than 3‰ range in individual values (−12.4‰ to −9.1‰), and, when corrected to present-day atmospheric δ^13^C values [107], suggest foraging in a relatively wet environment. Conversely, taxa sampled from Middle Eocene deposits of Northern Pakistan displayed a much wider range of δ^18^O values (21 to 27‰) and much higher enamel δ^13^C values (Fig. 5B,C).

Enamel δ^18^O values for the anthracobunids *Anthracobune pinfoldi* (23.5±1.2‰), *Anthracobune wardi* (25.7±1.8‰), and *Obergfellia occidentalis* (24.3‰) from the Kuldana Formation fall in between enamel δ^18^O values for aquatic taxa (archaeocetes, raoellids; 21 to 25‰) and terrestrial taxa (*Jhagirolophus*, 26.8‰; undescribed artiodactyl, 27.0±1.0‰) (Fig. 5B). These values are suggestive but not conclusive evidence of aquatic habits for these anthracobunids; the small number of terrestrial taxa available from these deposits (n = 2) limits the efficacy of our comparison. Enamel δ^13^C values for *A. pinfoldi* (−9.0±0.8‰), *A. wardi* (−9.6±0.5‰), and *O. occidentalis* (−8.9‰) are most similar to values for rhinos, tapirs, and other perissodactyls from these deposits (−9.9 to −8.7‰), suggesting that like these taxa, anthracobunids browsed on terrestrial C_3_ foliage. These two groups had much higher enamel δ^13^C values than freshwater-foraging archaeocetes (−14.8 to −9.0‰) and showed less variation than artiodactyls (mostly raoellids, −11.4 to −6.9‰), which may have had more flexible diets.

Two specimens of *A. wardi* collected from Middle Eocene deposits of northern India had low enamel δ^18^O values (21.3‰ and 21.8‰) that approached values for aquatic taxa, but also overlapped with values for terrestrial rhinocerotoids (20.6‰ to 24.7‰) from the same formation. These low enamel δ^18^O values are more consistent with a freshwater habitat for these individuals, but, again, low sample size complicates differentiating δ^18^O values for these specimens from δ^18^O values for taxa assumed to be fully terrestrial.

## Discussion

### Anthracobunids are Stem Perissodactyls

Anthracobunids have not previously been incorporated into taxonomically broad phylogenetic analyses of morphological data that incorporate the molecular evidence for placental supraordinal relationships. We tested for the possibility that Anthracobunidae and various Laurasian “condylarths” (phenacodontids, louisinids) are not members of Afrotheria by adding a number of living and extinct laurasiatherians to a morphological character matrix that has previously been employed to reconstruct afrotherian phylogeny [19], [35], [108]. We constrained relationships of extant taxa, based largely on the results of Meredith et al. [27], using the “molecular scaffold” technique proposed by Springer et al. [109]. We also employed several alternative constraints to obtain tree lengths for competing topologies.

Regardless of how ordered multistate characters were weighted, or whether Xenarthra was placed as the sister group of Afrotheria (i.e., the Atlantogenata hypothesis) or of Euarchontoglires + Laurasiatheria (the Exafroplacentalia hypothesis) in the molecular scaffold, parsimony analysis of the morphological data always placed anthracobunids within Laurasiatheria as members of Perissodactyla (Fig. 3). Surprisingly, members of the Order Desmostylia — which, like anthracobunids, have long been placed in Paenungulata — also grouped consistently with laurasiatherian perissodactyls rather than with afrotherian tethytheres, as previously suggested on the basis of similarities in tooth enamel by Ijiri and Kamei [110]. None of these clades were found in more than 50% of the bootstrap replicates, however. Parsimony analysis with all characters unordered and equally weighted consistently placed *Anthracobune* in Laurasiatheria with Perissodactyla, but the less well-known *Obergfellia* and *Cambaytherium* were “wild-card” taxa that either grouped with Desmostylia and Sirenia (within Afrotheria) or with Perissodactyla (within Laurasiatheria) across 658 equally parsimonious trees.

When continental geography is mapped onto the Adams consensus in Fig. 3, crown and advanced stem perissodactyls (including *Cambaytherium* and *Radinskya*) are optimized as having originated in Asia (Fig. 3), while the perissodactyl stem lineage is of ambiguous (but certainly Laurasian) continental origin. Later colonization of North America by crown perissodactyls was presumably facilitated by global warming at the Paleocene-Eocene boundary [111], [112]. Afrotheria is unambiguously of Afro-Arabian origin on the Adams topology, and the recently described Paleocene African “condylarth” *Ocepeia* [58] was placed as either a stem paenungulate or as a stem atlantogenatan, depending on whether Atlantogenata or Exafroplacentalia was constrained (respectively). The consistent recovery of the European louisinids *Paschatherium* and *Teilhardimys* as stem perissodactyls challenges the view that they are afrotherian macroscelideans [62].

Several studies have identified the early Paleogene Laurasian “condylarths” *Meniscotherium*, *Paschatherium*, *Phenacodus*, and *Teilhardimys* as members of Paenungulata or Macroscelidea [18], [60]–[64]. Some of the authors of these studies have in turn suggested that Afrotheria might not be of Afro-Arabian origin. The reliability of the analyses that place these Laurasian “condylarths” within Afrotheria has been called into question due to concerns that character and taxon sampling has potentially been inadequate for confident supraordinal placement of these and other fossil taxa [69], [113]. Our results have an important bearing on this issue, because the matrix analyzed here incorporates denser sampling of taxa (particularly within Afrotheria) than all previous studies. In our more detailed analyses, *Meniscotherium*, *Paschatherium*, *Phenacodus*, and *Teilhardimys* are consistently placed as stem perissodactyls across all assumption sets. This expanded clade of stem and crown perissodactyls was reconstructed as having been of either North American, Asian, or European origin. This biogeographic reconstruction is undoubtedly greatly oversimplified given that numerous allied taxa that were distributed across Laurasia were not included in the analysis, and Carnivora and Artiodactyla are only represented by three taxa. Afrotheria, Paenungulata, Tethytheria, Afroinsectivora, and Afrosoricida were reconstructed as having been of Afro-Arabian origin across all assumption sets. Experimental grafting of embrithopod genera from the Eocene of Turkey and Romania [114], [115] to the base of Embrithopoda does not alter this biogeographic reconstruction.

### Reconstructing the Ancient Habitat and Diet of Anthracobunids

Some early tethytheres are thought to have had a semi-aquatic lifestyle (proboscideans [81], [116], desmostylians [117], sirenians [104], [118]–[120]), but no evidence suggests that stem perissodactyls occupied an aquatic habitat. To reconstruct the ancient habitat preferences of anthracobunids, we analyzed bone geometry of the postcranial skeleton as well as stable isotope values within tooth enamel.

We quantified the midshaft cross-sectional geometries of limb and rib bones of anthracobunids and other ungulates. *Anthracobune* limb bones displayed bones that were more hyperostotic and therefore more compacted compared to most extant ungulates. Exceptions included extant taxa that are known to frequently swim or wallow in shallow water habitats (i.e., *Hippopotamus* and *Rhinoceros*) [121], and fossil Eocene cetaceans that are thought to be semi-aquatic and also occupy shallow water habitats (i.e., pakicetid and remingtonocetids) (Fig. 4). Thus, anthracobunids display a variety of skeletal modifications consistent with taxa that exploit shallow-water habitats, including thickened postcranial elements and widened elements of the autopodium, in addition to large body size.

We also studied the oxygen and carbon isotope composition of structural carbonate in tooth enamel [75], [106] for anthracobunids and coeval taxa (Fig. 5). The anthracobunids recovered from the Kuldana formation (*Anthracobune* and *Obergfellia*) displayed enamel δ^18^O values suggesting some evidence of aquatic habits, and enamel δ^13^C values showed they fed on land. Anthracobunids recovered from northern India displayed enamel δ^18^O values consistent with occupation of freshwater habitats. Although all sampled anthracobunids may have had some aquatic preferences, stable isotopic evidence alone is inconclusive as there is overlap in range of isotopic values between terrestrial and aquatic taxa.

Taken together, skeletal and isotopic evidence are most consistent with an interpretation of anthracobunids (especially *Anthracobune*) as having had ecological preferences similar to those of modern rhinos and tapirs, but not exhibiting the same degree of commitment to aquatic habits as *Hippopotamus* [84], [122]. Most rhinos and tapirs have a restricted range near permanent bodies of water in which they frequently wallow and wade [121], and obtain most of their drinking water [84], [122]. Modern rhinos may be a good model for the anthracobunid lifestyle as both display thickened limbs and intermediate oxygen isotope values relative to *Hippopotamus* and terrestrial ungulates.

Stable isotopic evidence also showed mammal fossils sampled from Early Eocene localities (Fig. 5C) occupied and foraged within a wet and forested habitat. Conversely, taxa sampled from Middle Eocene deposits of Northern Pakistan (Fig. 5B,C) indicated shifts in the climate toward increased aridity. This climate change was most likely caused by the northward movement of the Indian plate, which pushed these areas out of an equatorial zone of high precipitation in the Middle Eocene [123], [124]. Vegetation would have opened up, and shifted to a drier, tropical forest or savanna habitat.

## Conclusions

Based on new dental, cranial, and postcranial material, our phylogenetic analysis is the first to suggest that anthracobunids are stem members of the laurasiatherian order Perissodactyla, rather than members of the afrotherian clades Tethytheria, Proboscidea, or Sirenia. The strictly Asian distribution of anthracobunids is consistent with the isolation of afrotherian and laurasiatherian clades through the early Paleogene [19], [113], while the phylogenetic result is consistent with the recent recovery of very primitive basal stem proboscideans such as *Phosphatherium* [125] and *Eritherium* [18], from the early Eocene and Paleocene of Africa. Analyses of postcranial bones and stable isotopes also indicate that anthracobunids were large and lumbering, and suggest at least some taxa spent time in the water similar to modern rhinos, and that all known anthracobunids fed on land. Our results therefore identify an Old World radiation of large, non-cursorial, partly aquatic perissodactyls that convergently came to occupy a basal tethythere-like niche on the northern coast of the Tethys Sea.

One of the more intriguing possibilities raised by our analysis is that the order Desmostylia, which has always been restricted to the Pacific Rim and has never been found in Afro-Arabia, might be aligned with perissodactyls rather than paenungulates, and that this clade might trace its origin back to south Asian anthracobunids, cambaytheres, or both.

This study also provided further resolution as to the placement of Paleocene *Radinskya* [36] [38] and early Eocene *Cambaytherium* as stem perissodactyls. Confusion surrounding dental similarities of *Cambaytherium* with perissodactyls and anthracobunids could be resolved by the placement of cambaytheriids and anthracobunids as closely related sister taxa to crown Perissodactyla, thereby indicating that similarities to tethytheres are entirely due to convergence.

## Supporting Information

Figure S1 **Mandibles of anthracobunids.** (*A*) *Anthracobune pinfoldi* (HGSP-97106) with p4, m2-3 in lateral view (m3 third lobe is missing). Mandible of *Obergfellia occidentalis* (H-GSP 96149) in (*B*) lateral view, and (*C*) superior view. Mandible of a juvenile of *Anthracobune wardi* (H-GSP 30349) with deciduous dp3-m1 in (*D*) lateral view, and (*E*) superior view. Scale bar is 1 cm in length.
(TIF)

Figure S2 **Strict consensus trees derived from primary analyses of the morphological data set constrained to fit the (A,B) Atlantogenata and (C,D) Exafroplacentalia constraints, with transitions between polymorphic and “fixed” states in ordered morphoclines weighted as either 0.5 steps (A and C) or 1.0 (B and D).**
(PDF)

Figure S3 **Strict consensus trees derived from parsimony analyses of the morphological data (transitions between polymorphic and “fixed” states in ordered morphoclines weighted as 0.5 steps) with the following alternative constraints: A) Anthracobunidae constrained to join Paenungulata; B) Desmostylia constrained to join Paenungulata; C)** ***Eritherium*** **and** ***Phosphatherium*** **constrained to be stem proboscideans (cf. Gheerbrant 2012); and D)** ***Cambaytherium*** **constrained to join anthracobunids to the exclusion of crown perissodactyls.**
(PDF)

Figure S4 **Strict consensus trees derived from parsimony analyses of the morphological data (transitions between polymorphic and “fixed” states in ordered morphoclines weighted as 1.0 steps) with the following alternative constraints: A) Anthracobunidae constrained to join Paenungulata; B) Desmostylia constrained to join Paenungulata; C)** ***Eritherium*** **and** ***Phosphatherium*** **constrained to be stem proboscideans (cf. Gheerbrant 2012); and D)** ***Cambaytherium*** **constrained to join anthracobunids to the exclusion of crown perissodactyls.**
(PDF)

Figure S5 **Strict consensus trees derived from parsimony analyses of the morphological data (all characters unordered) with the following alternative constraints: A) Anthracobunidae constrained to join Paenungulata; B)** ***Eritherium*** **and** ***Phosphatherium*** **constrained to be stem proboscideans (cf. Gheerbrant 2012).**
(PDF)

Table S1 **Unambiguous character support for the monophyly of Anthracobunidae (** ***Anthracobune*** **+** ***Obergfellia*** **), given the Adams consensus tree depicted in** **Figure 3** **of the main text.**
(PDF)

Table S2 **Unambiguous character support for the monophyly of a clade containing Anthracobunidae,** ***Cambaytherium*** **,** ***Hallensia*** **,** ***Radinskya*** **, and “advanced” perissodactyls, given the Adams consensus tree depicted in** **Figure 3** **of the main text.**
(PDF)

Table S3 **Parsimony reconstructions for continental biogeography, optimized onto the Adams consensus tree depicted in** **Figure 3** **of the main text.**
(PDF)

Table S4 **Tree lengths for alternative topological hypotheses, number of steps longer than the less constrained topology, and** ***P*** **values from non-parametric Templeton tests (first tree of each tree file only), with steps between ‘fixed’ and ‘polymorphic’ states in ordered multistates weighted as 0.5. Constrained topologies: 1) Molecular scaffold alone; 3) Anthracobunidae constrained to join Paenungulata; 4) Desmostylia constrained to join Paenungulata; 5)** ***Eritherium*** **and** ***Phosphatherium*** **constrained to be stem proboscideans (cf. Gheerbrant 2012); and 6)** ***Cambaytherium*** **constrained to join anthracobunids to the exclusion of crown perissodactyls (See Fig. S3 for topologies).**
(PDF)

Table S5 **Tree lengths for alternative topological hypotheses, number of steps longer than the less constrained topology, and** ***P*** **values from non-parametric Templeton tests (first tree of each tree file only), with steps between ‘fixed’ and ‘polymorphic’ states in ordered multistates weighted as 1.0. Constrained topologies: 1) Molecular scaffold alone; 3) Anthracobunidae constrained to join Paenungulata; 4) Desmostylia constrained to join Paenungulata; 5)** ***Eritherium*** **and** ***Phosphatherium*** **constrained to be stem proboscideans (cf. Gheerbrant 2012); and 6)** ***Cambaytherium*** **constrained to join anthracobunids to the exclusion of crown perissodactyls (See Fig. S4 for topologies).**
(PDF)

Table S6 **Tree lengths for alternative topological hypotheses, number of steps longer than the less constrained topology, and** ***P*** **values from non-parametric Templeton tests, with all characters unordered and equally weighted.** Constrained topologies: 1) Molecular scaffold alone; 3) Anthracobunidae constrained to join Paenungulata; and 5) *Eritherium* and *Phosphatherium* constrained to be stem proboscideans (cf. Gheerbrant 2012). (See Fig. S5 for topologies).
(PDF)

Table S7 **Taxonomy, specimen number, bone, and bone compactness values for limb and rib bones analyzed in this study.**
(PDF)

Table S8 **Taxonomy, specimen number, tooth identification, and summary statistics of stable isotope values of fossil enamel samples used in this study.**
(PDF)

Table S9 **Taxonomy, specimen number, tooth identification, and stable isotope values of fossil enamel samples used in this study.**
(PDF)

Matrix Nexus S1 **Nex file.**
(NEX)

Matrix Tree S1 **Tre file.**
(TRE)

Text S1 **Additional taxonomic description.**
(DOCX)
