# Supplementary figures and images for: Anthracobunids from the Middle Eocene of India and Pakistan Are Stem Perissodactyls
Source: PLoS One. 2014 Oct 8;9(10):e109232. doi: 10.1371/journal.pone.0109232 (PMC4189980; doi:10.1371/journal.pone.0109232)

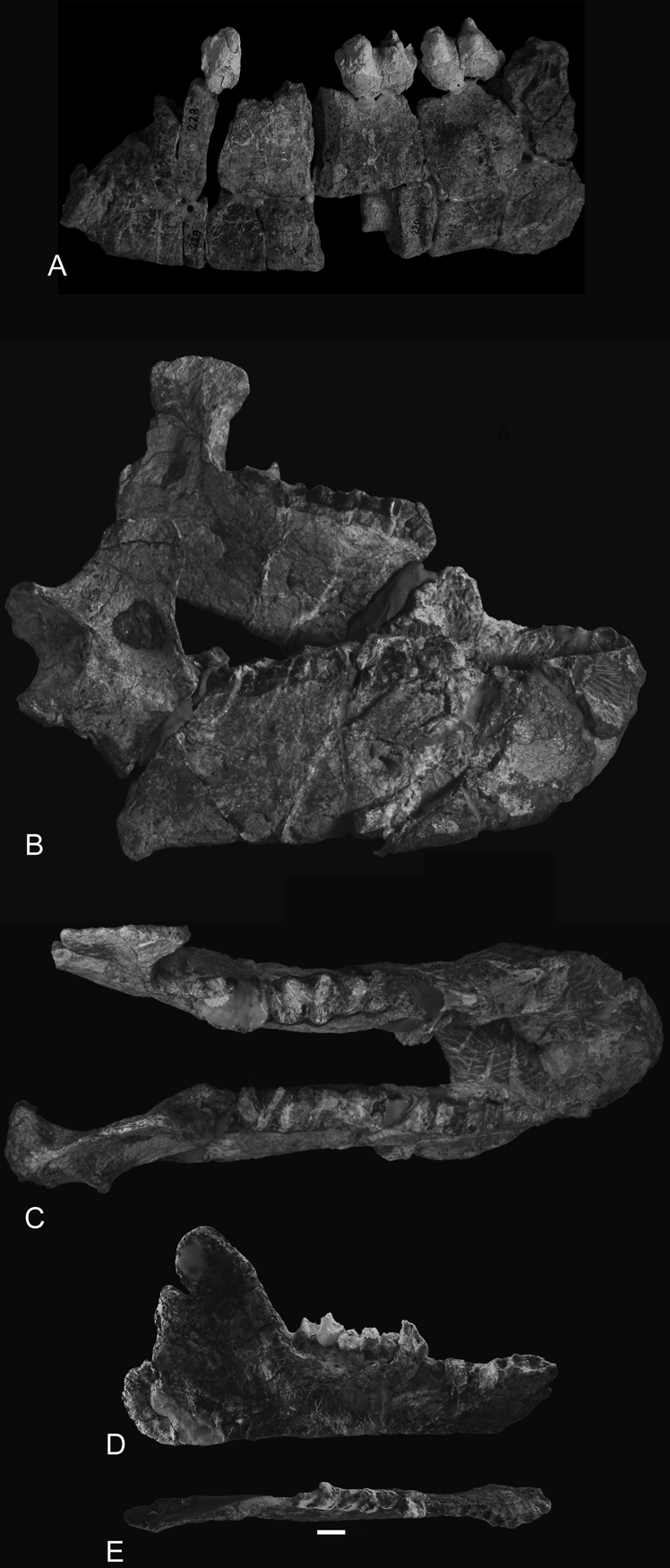

Supplement: Figure S1 — Mandibles of anthracobunids. (A) Anthracobune pinfoldi (HGSP-97106) with p4, m2-3 in lateral view (m3 third lobe is missing). Mandible of Obergfellia occidentalis (H-GSP 96149) in (B) lateral view, and (C) superior view. Mandible of a juvenile of Anthracobune wardi (H-GSP 30349) with deciduous dp3-m1 in (D) lateral view, and (E) superior view. Scale bar is 1 cm in length. (TIF) [file pone.0109232.s001.tif]
