# Supplementary material for: Anthracobunids from the Middle Eocene of India and Pakistan Are Stem Perissodactyls
Source: PLoS One. 2014 Oct 8;9(10):e109232. doi: 10.1371/journal.pone.0109232 (PMC4189980; doi:10.1371/journal.pone.0109232)
Supplement: Figure S2 — Strict consensus trees derived from primary analyses of the morphological data set constrained to fit the (A,B) Atlantogenata and (C,D) Exafroplacentalia constraints, with transitions between polymorphic and “fixed” states in ordered morphoclines weighted as either 0.5 steps (A and C) or 1.0 (B and D). (PDF) [file pone.0109232.s002.pdf]

Primary topologies, Atlantogenata constraint

Primary topologies, Exafroplacentalia constraint

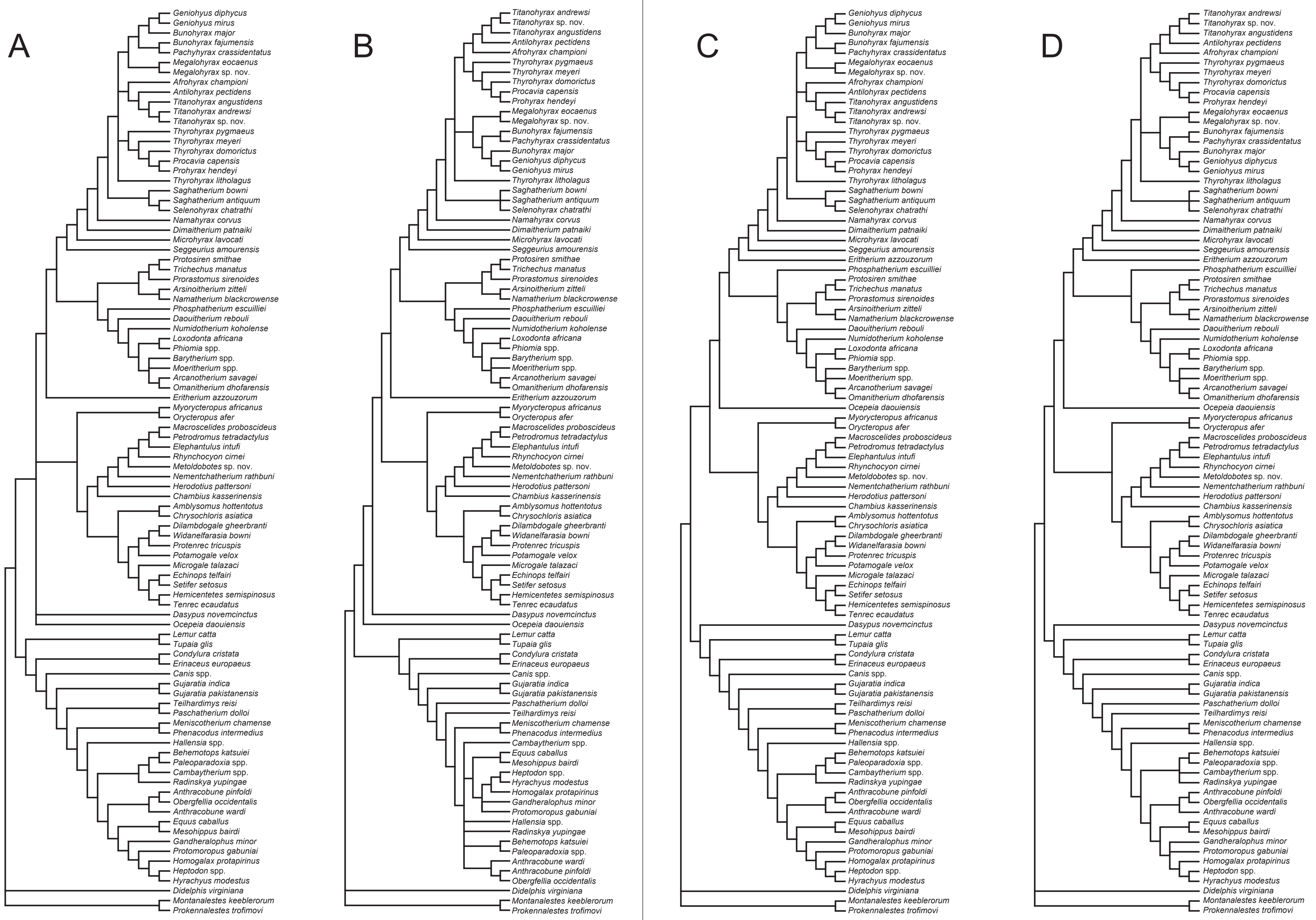

Constraint 1 (A1) with 0.5 weighting of ordered multistates with intermediate states for polymorphisms. Tree length = **3704.5**.

Constraint 1 (A2) with no weighting of ordered multistates with intermediate states for polymorphisms. Tree length = **5758**.

Constraint 2 (E1) with 0.5 weighting of ordered multistates with intermediate states for polymorphisms. Tree length = **3716**

Constraint 2 (E2) with no weighting of ordered multistates with intermediate states for polymorphisms. Tree length = **5773**.
