# Supplementary material for: Anthracobunids from the Middle Eocene of India and Pakistan Are Stem Perissodactyls
Source: PLoS One. 2014 Oct 8;9(10):e109232. doi: 10.1371/journal.pone.0109232 (PMC4189980; doi:10.1371/journal.pone.0109232)
Supplement: Figure S3 — Strict consensus trees derived from parsimony analyses of the morphological data (transitions between polymorphic and “fixed” states in ordered morphoclines weighted as 0.5 steps) with the following alternative constraints: A) Anthracobunidae constrained to join Paenungulata; B) Desmostylia constrained to join Paenungulata; C) Eritherium and Phosphatherium constrained to be stem proboscideans (cf. Gheerbrant 2012); and D) Cambaytherium constrained to join anthracobunids to the exclusion of crown perissodactyls. (PDF) [file pone.0109232.s003.pdf]

Alternative constraints, with steps between ‘fixed’ and ‘polymorphic’ states in ordered multistates weighted as 0.5

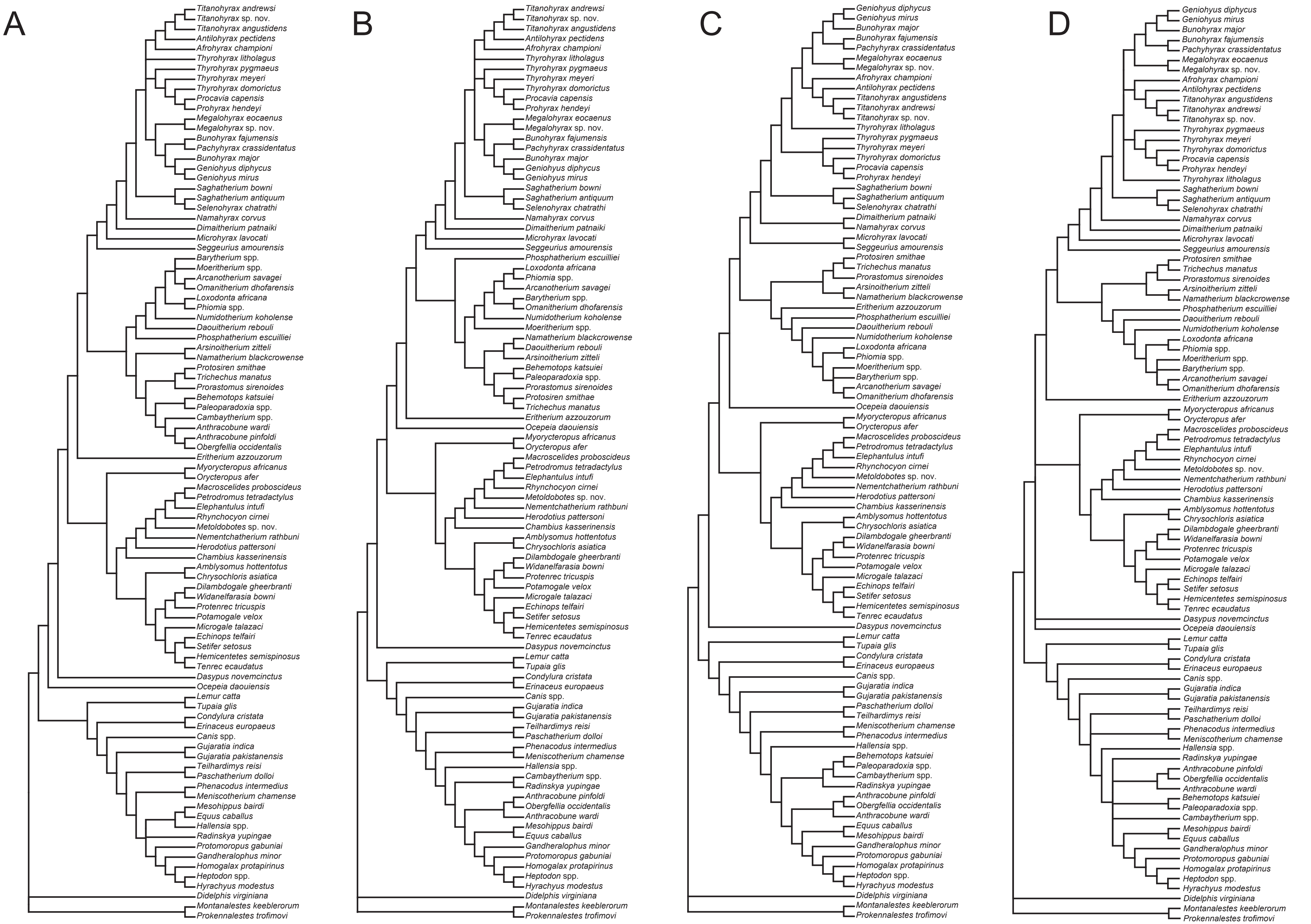

Constraint 3: Anthracobunidae constrained to join Paenungulata. Tree length = **3711** (+6.5 steps).

Constraint 4: Desmostylia constrained to join Paenungulata. Tree length = **3702** (+ 3.5 steps).

Constraint 5: *Eritherium* and *Phosphatherium* constrained to be stem proboscideans. Tree length = **3710** (+5.5 steps).

Constraint 6: *Cambaytherium* constrained to join anthracobunids to the exclusion of crown perissodactyls. Tree length = **3707.5** (+ 3 steps).
