# Supplementary material for: Anthracobunids from the Middle Eocene of India and Pakistan Are Stem Perissodactyls
Source: PLoS One. 2014 Oct 8;9(10):e109232. doi: 10.1371/journal.pone.0109232 (PMC4189980; doi:10.1371/journal.pone.0109232)
Supplement: Figure S5 — Strict consensus trees derived from parsimony analyses of the morphological data (all characters unordered) with the following alternative constraints: A) Anthracobunidae constrained to join Paenungulata; B) Eritherium and Phosphatherium constrained to be stem proboscideans (cf. Gheerbrant 2012). (PDF) [file pone.0109232.s005.pdf]

Alternative constraints, with all characters unordered and equally weighted

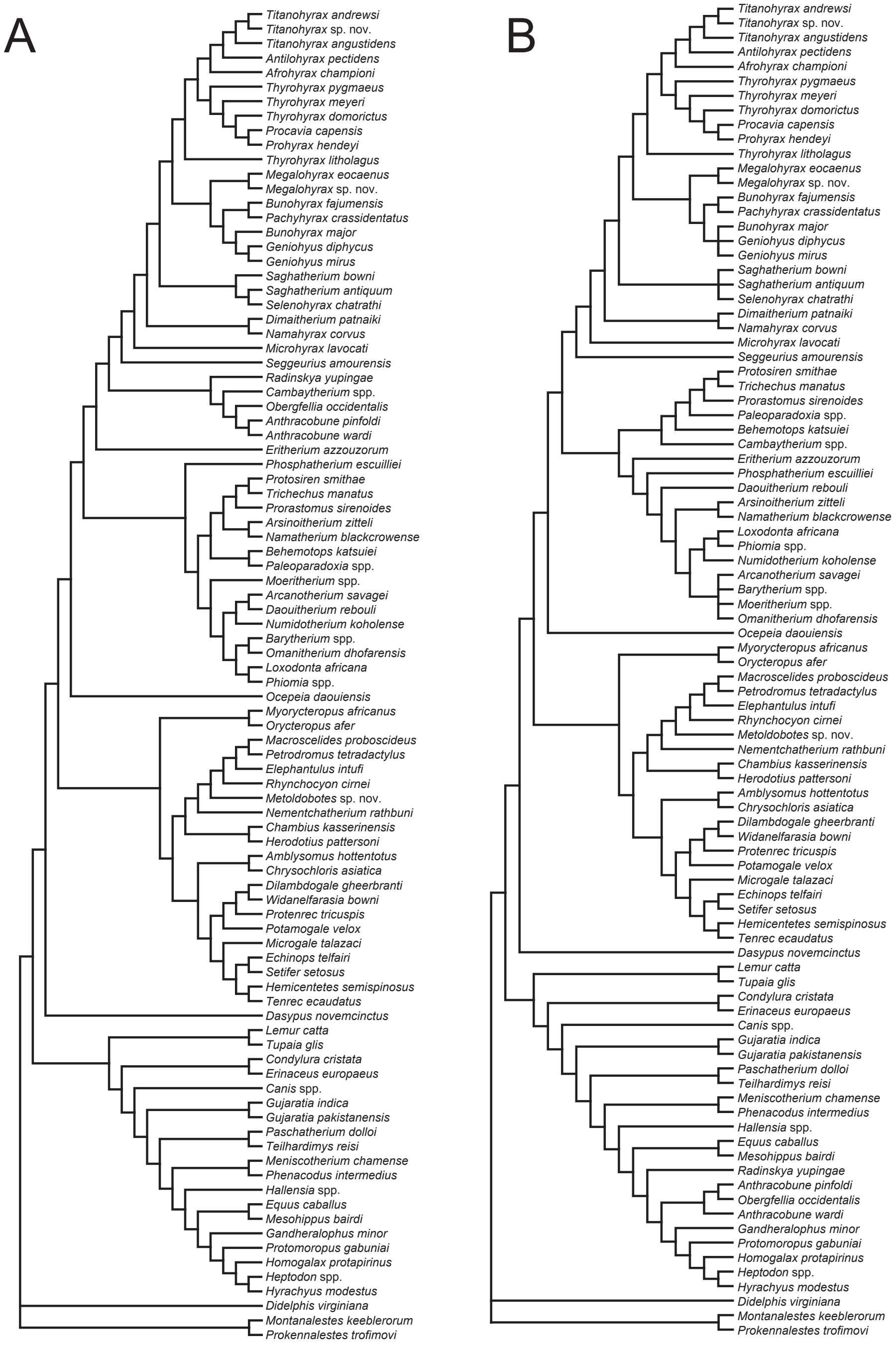

Constraint 3: Anthracobunidae constrained to join Paenungulata. Tree length = **3662** (+2 steps).

Constraint 5: *Eritherium* and *Phosphatherium* constrained to be stem proboscideans. Tree length = **3669** (+9 steps).
