# Supplementary material for: Anthracobunids from the Middle Eocene of India and Pakistan Are Stem Perissodactyls
Source: PLoS One. 2014 Oct 8;9(10):e109232. doi: 10.1371/journal.pone.0109232 (PMC4189980; doi:10.1371/journal.pone.0109232)
Supplement: Table S1 — Unambiguous character support for the monophyly of Anthracobunidae ( Anthracobune + Obergfellia ), given the Adams consensus tree depicted in Figure 3 of the main text. (PDF) [file pone.0109232.s006.pdf]

Table S1. Unambiguous character support for the monophyly of Anthracobunidae (*Anthracobune* + *Obergfellia*), given the consensus tree depicted in Figure 3 of the main text.

| **Character number and state change** | **Description of character state change** |
|---|---|
| 17 (4 => 1) | Diastema between canine and adjacent premolar is present and is much longer than the mesiodistal length of the lower canine => Polymorphic expression (no diastema is present/diastema is present, but its mesiodistal length is half of, or equal to, the mesiodistal length of the lower canine) |
| 26 (0 => 1) | $P_2$ metaconid absent => Polymorphic expression ($P_2$ metaconid absent/$P_2$ metaconid present but small relative to protoconid) |
| 29 (0 => 4) | $P_3$ paraconid absent or very small => $P_3$ paraconid large and distinct |
| 32 (0 => 1) | $P_3$ entoconid absent => Polymorphic expression ($P_3$ entoconid absent/$P_3$ present but smaller than hypoconid) |
| 38 (0 => 1) | $P_4$ premetacristid absent or indistinct => Polymorphic expression ($P_4$ premetacristid absent or indistinct/$P_4$ premetacristid present, trenchant) |
| 40 (2 => 1) | $P_4$ hypoconid greater than half the height of the protoconid => $P_4$ hypoconid less than half the height of the protoconid |
| 43 (4 => 3) | Hypoconid buccally placed on $P_4$ => Polymorphic expression (hypoconid buccally placed on $P_4$/hypoconid centrally placed on $P_4$) |
| 46 (2 => 1) | $P_4$ talonid is approximately equal in width to the trigonid => Polymorphic expression ($P_4$ talonid is approximately equal in width to the trigonid/$P_4$ talonid is narrower than trigonid) |
| 71 (0 => 2) | $M_2$ hypoflexid shallow, most of the space distal to postcristid and mesial to the hypoconid is composed of talonid basin defined labially by cristid obliqua => $M_2$ hypoflexid deep, most of the space distal to the postcristid and mesial to the hypoconid is labially open for occlusion with paracone |
| 74 (2 => 0) | Cristid obliqua-hypocristid junction on $M_{1-2}$ not distinctly "v"-shaped, or is gently rounded => cristid obliqua on $M_{1-2}$ meets hypoconid at a sharp angle, forming a distinct "v" |
| 81 (4 => 6) | $M_3$ hypoconulid large, lobate, unicuspid => $M_3$ hypoconulid large, lobate, two or more cusps present |
| 86 (4 => 3) | Enamel present and smooth => Polymorphic expression (enamel present and smooth/enamel present and crenulated) |
| 118 (4 => 2) | $P^3$ metacone present, distinct, and differentiated from paracone => $P^3$ metacone present, but not well-differentiated and closely appressed to paracone |
| 131 (4 => 2) | $P^4$ metacone present, distinct, and differentiated from paracone => $P^4$ metacone present, but not well-differentiated and closely appressed to paracone |

| | |
|---|---|
| 165 (0 => 1) | Buccal cleft between paracone and metacone on $M^2$ (ectoflexus) absent => Polymorphic expression (buccal cleft between paracone and metacone on $M^2$ (ectoflexus) absent/present) |
| 287 (1 => 3) | Polymorphic expression (ascending ramus of mandible posteriorly inclined/vertical with respect to occlusal plane) => Polymorphic expression (ascending ramus of mandible vertical with respect to occlusal plane/anteriorly inclined) |
