# Supplementary material for: Anthracobunids from the Middle Eocene of India and Pakistan Are Stem Perissodactyls
Source: PLoS One. 2014 Oct 8;9(10):e109232. doi: 10.1371/journal.pone.0109232 (PMC4189980; doi:10.1371/journal.pone.0109232)
Supplement: Table S2 — Unambiguous character support for the monophyly of a clade containing Anthracobunidae, Cambaytherium , Hallensia , Radinskya , and “advanced” perissodactyls, given the Adams consensus tree depicted in Figure 3 of the main text. (PDF) [file pone.0109232.s007.pdf]

Table S2. Unambiguous character support for the monophyly of a clade containing Anthracobunidae, *Cambaytherium*, *Hallensia*, *Radinskya*, and “advanced” perissodactyls, given the Adams consensus tree depicted in Figure 3 of the main text.

**Character number and state change**	**Description of character state change**
51 (0 => 4)	M_1 area = 0-50 mm^2 => M_1 area 101-150 mm^2
76 (0 => 1)	No crests connecting hypoconid and entoconid on lower molars => incipient crests connecting entoconid and hypoconid are present on lower molars, but are poorly developed and depressed (low)
81 (3 => 4)	Polymorphic condition (M_3 hypoconulid reduced, unicuspid/M_3 hypoconulid lobe forms a large, lobate structure) => M_3 hypoconulid lobe forms a large, lobate structure
82 (3 => 0)	Polymorphic expression (M_3 area is subequal to M_2 area/M_3 area is less than M_2 area => M_3 area is greater than M_2 area
145 (3 => 4)	Polymorphic condition (mesial cingulum on M^{1-2} is well-developed on the mesial aspect of protocone, but is absent labially/mesial cingulum on M^{1-2} is complete, well-defined across all or most of the mesial aspect of the tooth) => mesial cingulum on M^{1-2} is complete, well-defined across all or most of the mesial aspect of the tooth
147 (1 => 0)	Polymorphic condition (postprotocristae on M^{1-2} absent/ postprotocristae on M^{1-2} weak) => postprotocristae on M^{1-2} absent
161 (2 => 0)	M^{1-2} paraconules present and distinct => M^{1-2} paraconules large and inflated
191 (4 => 6)	Four sacral vertebrae => five sacral vertebrae
227 (0 => 1)	Pollex present => pollex absent or vestigial
265 (0 => 2)	Navicular facet of astragalus convex => navicular facet of astragalus concavoconvex
335 (1 => 0)	Orbitotemporal foramina widely exposed laterally => orbitotemporal foramina posteriorly crowded
