# Supplementary material for: Anthracobunids from the Middle Eocene of India and Pakistan Are Stem Perissodactyls
Source: PLoS One. 2014 Oct 8;9(10):e109232. doi: 10.1371/journal.pone.0109232 (PMC4189980; doi:10.1371/journal.pone.0109232)
Supplement: Table S3 — Parsimony reconstructions for continental biogeography, optimized onto the Adams consensus tree depicted in Figure 3 of the main text. (PDF) [file pone.0109232.s008.pdf]

Table S3. Parsimony reconstructions for continental biogeography

Assumption set				Adams
A1	A2	E1	E2	
Anthracobunidae+Cambaytheriidae+"crown Perissodactyla"+*Radinskya*+*Hallensia*				
Asia/NA/Eur	Asia	Asia/NA/Eur	Europe	Asia/Eur
Anthracobunidae+Cambaytheriidae+"crown Perissodactyla"+*Radinskya*				
Asia	Not present	Asia	Asia	Not present
Equoidea-Chalicotheroidea-Tapiroidea (="crown Perissodactyla")				
Asia	Asia	Asia	Asia	Not present
Afrotheria				
Afro-Arabia	Afro-Arabia	Afro-Arabia	Afro-Arabia	Afro-Arabia

NA = North America, Eur = Europe
