# Supplementary material for: Anthracobunids from the Middle Eocene of India and Pakistan Are Stem Perissodactyls
Source: PLoS One. 2014 Oct 8;9(10):e109232. doi: 10.1371/journal.pone.0109232 (PMC4189980; doi:10.1371/journal.pone.0109232)
Supplement: Table S4 — Tree lengths for alternative topological hypotheses, number of steps longer than the less constrained topology, and P values from non-parametric Templeton tests (first tree of each tree file only), with steps between ‘fixed’ and ‘polymorphic’ states in ordered multistates weighted as 0.5. Constrained topologies: 1) Molecular scaffold alone; 3) Anthracobunidae constrained to join Paenungulata; 4) Desmostylia constrained to join Paenungulata; 5) Eritherium and Phosphatherium constrained to be stem proboscideans (cf. Gheerbrant 2012); and 6) Cambaytherium constrained to join anthracobunids to the exclusion of crown perissodactyls (See Fig. S3 for topologies). (PDF) [file pone.0109232.s009.pdf]

Table S4. Tree lengths for alternative topological hypotheses, number of steps longer than the less constrained topology, and *P* values from non-parametric Templeton tests (first tree of each tree file only), with steps between ‘fixed’ and ‘polymorphic’ states in ordered multistates weighted as 0.5. Constrained topologies: 1) Molecular scaffold alone; 3) Anthracobunidae constrained to join Paenungulata; 4) Desmostylia constrained to join Paenungulata; 5) *Eritherium* and *Phosphatherium* constrained to be stem proboscideans (cf. Gheerbrant 2012); and 6) *Cambaytherium* constrained to join anthracobunids to the exclusion of crown perissodactyls (See Fig. S5 for topologies).

Constraint	Length	Length difference	*P*	Anthracobunid sister taxon	Position of Desmostylia
1	3704.5	-	-	Crown Perissodactyla	Stem Perissodactyla
3	3711	6.5	0.7633	*Cambaytherium*	Stem Sirenia
4	3708	3.5	0.8072	Crown Perissodactyla	Stem Sirenia
5	3710	5.5	0.3104	Crown Perissodactyla	Stem Perissodactyla
6	3707.5	3	0.7466	Perissodactyla, unresolved	Perissodactyla, unresolved
