# Supplementary material for: Anthracobunids from the Middle Eocene of India and Pakistan Are Stem Perissodactyls
Source: PLoS One. 2014 Oct 8;9(10):e109232. doi: 10.1371/journal.pone.0109232 (PMC4189980; doi:10.1371/journal.pone.0109232)
Supplement: Table S5 — Tree lengths for alternative topological hypotheses, number of steps longer than the less constrained topology, and P values from non-parametric Templeton tests (first tree of each tree file only), with steps between ‘fixed’ and ‘polymorphic’ states in ordered multistates weighted as 1.0. Constrained topologies: 1) Molecular scaffold alone; 3) Anthracobunidae constrained to join Paenungulata; 4) Desmostylia constrained to join Paenungulata; 5) Eritherium and Phosphatherium constrained to be stem proboscideans (cf. Gheerbrant 2012); and 6) Cambaytherium constrained to join anthracobunids to the exclusion of crown perissodactyls (See Fig. S4 for topologies). (PDF) [file pone.0109232.s010.pdf]

Table S5. Tree lengths for alternative topological hypotheses, number of steps longer than the less constrained topology, and *P* values from non-parametric Templeton tests (first tree of each tree file only), with steps between ‘fixed’ and ‘polymorphic’ states in ordered multistates weighted as 1.0. Constrained topologies: 1) Molecular scaffold alone; 3) Anthracobunidae constrained to join Paenungulata; 4) Desmostylia constrained to join Paenungulata; 5) *Eritherium* and *Phosphatherium* constrained to be stem proboscideans (cf. Gheerbrant 2012); and 6) *Cambaytherium* constrained to join anthracobunids to the exclusion of crown perissodactyls (See Fig. S6 for topologies).

Constraint	Length	Length difference	*P*	Anthracobunid sister taxon	Position of Desmostylia
1	5758	-	-	Hippomorpha	Stem Perissodactyla
3	5772	14	0.6280	*Cambaytherium*	Stem Sirenia
4	5766	8	0.5457	Perissodactyla, unresolved	Stem Sirenia
5	5773	15	0.2440	Crown Perissodactyla	Stem Perissodactyla
6	5762	4	0.7880	*Cambaytherium*	Stem Perissodactyla
