# Supplementary material for: Anthracobunids from the Middle Eocene of India and Pakistan Are Stem Perissodactyls
Source: PLoS One. 2014 Oct 8;9(10):e109232. doi: 10.1371/journal.pone.0109232 (PMC4189980; doi:10.1371/journal.pone.0109232)
Supplement: Table S6 — Tree lengths for alternative topological hypotheses, number of steps longer than the less constrained topology, and P values from non-parametric Templeton tests, with all characters unordered and equally weighted. Constrained topologies: 1) Molecular scaffold alone; 3) Anthracobunidae constrained to join Paenungulata; and 5) Eritherium and Phosphatherium constrained to be stem proboscideans (cf. Gheerbrant 2012). (See Fig. S5 for topologies). (PDF) [file pone.0109232.s011.pdf]

Table S6. Tree lengths for alternative topological hypotheses, number of steps longer than the less constrained topology, and *P* values from non-parametric Templeton tests, with all characters unordered and equally weighted. Constrained topologies: 1) Molecular scaffold alone; 3) Anthracobunidae constrained to join Paenungulata; and 5) *Eritherium* and *Phosphatherium* constrained to be stem proboscideans (cf. Gheerbrant 2012). (See Fig. S7 for topologies)

Constraint	Length	Length difference	*P*	Anthracobunid sister taxon	Position of Desmostylia
1	3660	-	-	Tapiromorpha*	Stem Sirenia
3	3662	2	0.7237	*Cambaytherium*/ *Radinskya*	Stem Sirenia
5	3669	9	0.3704	Tapiromorpha*	Stem Sirenia
