# Supplementary material for: Anthracobunids from the Middle Eocene of India and Pakistan Are Stem Perissodactyls
Source: PLoS One. 2014 Oct 8;9(10):e109232. doi: 10.1371/journal.pone.0109232 (PMC4189980; doi:10.1371/journal.pone.0109232)
Supplement: Table S7 — Taxonomy, specimen number, bone, and bone compactness values for limb and rib bones analyzed in this study. (PDF) [file pone.0109232.s012.pdf]

Summary Table 7: Compactness values of the long bone midshafts and ribs of fossil and extant mammals. Compactness values for long bone midshafts are shown in Figure 4. If sample sizes were greater than one for a given taxon (e.g., *Odocoileus*), averages were compared between taxa.

Taxon	ID Number	Humerus	Radius	Femur	Tibia	Rib
**Eocene Cetaceans and Sirenian**						
Ichthyolestes	HGSP 30345			0.95		
Ichthyolestes	HGSP 30357				0.93	
Ichthyolestes	No number	0.95				
Ichthyolestes	HGSP 96168					0.902
Kutchicetus	IITR-SB 2647	0.96			0.94	
Andrewsiphius	IITR-SB 2871			0.94		
*Pakicetus**	HGSP 96602					0.685
*Gaviacetus**	IITR-SB 2870					0.942
*Georgiacetus**	GSM-350					0.918
*Remingtonocetus**	IITR-SB 2653					0.826
Sirenian*	Ratipur 04-29					0.986
**Extant Artiodactyls**						
Hippopotamus amphibius	AMNH 15898	0.74		0.88	0.92	
Potamochoerus porcus	USNM 164542			0.68	0.8	
Odocoileus virginianus	USNM 396283	0.56		0.55	0.67	
Odocoileus virginianus	USNM 254653	0.63		0.68	0.76	
Odocoileus virginianus	USNM 254652	0.62		0.6	0.73	
*Odocoileus virginianus**						0.784
*Odocoileus virginianus**						0.542
*Odocoileus virginianus**						0.509
Kobus megaceros leucotis	AMNH 82135	0.49		0.61	0.69	
Kobus megaceros	USNM 164777				0.64	
Kobus leche	USNM 254927	0.58		0.69	0.77	
Kobus leche	AMNH 70010	0.61		0.66	0.78	
Hydropotes inermis	USNM 304664	0.73		0.63	0.76	
Bubalis bubalis	AMNH 54765	0.6		0.72	0.79	
Bos taurus	USNM 277262	0.42		0.66	0.66	
Axis axis	USNM 122532	0.66		0.54	0.64	
Alces americanus	USNM 275127	0.54		0.47	0.64	
**Paleogene Perissodactyls**						
Anthracobune	HGSP 96196		0.90			
Anthracobune	HGSP 97106		0.85			0.909, 0.838
Anthracobune	HGSP 96454		0.96			
Triplopus	CM 10190		0.79		0.76	
Hyracotherium	CM 62391	0.7				
Hyracotherium	CM 1180				0.75	
Mesohippus	CM 996	0.68	0.66		0.71	
Mesohippus	CM 3562			0.65	0.69	
**Extant Perissodactyls**						
Equus caballus	AMNH 14131	0.52	0.72	0.43	0.76	
Equus burchelli	AMNH 54287	0.55	0.79	0.57	0.80	
Tapirus terrestris	AMNH 14690	0.56	0.76	0.56	0.83	
Rhinoceros unicornis	AMNH 54454	0.64	0.84	0.75	0.85	
**Extant Mammals**						
*Canis domesticus**						0.716
*Enhydra lutris**						0.962
*Equus caballus**						0.596

*Lutra canadensis**						0.671
*Lutra canadensis**						0.628
*Lutra canadensis**						0.66
*Lutra canadensis**						0.582
*Specimens taken from Gray et al., 2007						
