# Supplementary material for: Anthracobunids from the Middle Eocene of India and Pakistan Are Stem Perissodactyls
Source: PLoS One. 2014 Oct 8;9(10):e109232. doi: 10.1371/journal.pone.0109232 (PMC4189980; doi:10.1371/journal.pone.0109232)
Supplement: Table S8 — Taxonomy, specimen number, tooth identification, and summary statistics of stable isotope values of fossil enamel samples used in this study. (PDF) [file pone.0109232.s013.pdf]

Supplementary Table 8: Taxa, age, region of provenance, inferred habitat (as based on stable isotopes), and summary statistics of Carbon- and Oxygen isotope composition of fossil tooth enamels of mammals from India and Pakistan.

**Taxon**	**Inferred Habitat**	**N**	**Mean δ^{13}C ± SD***		**Mean δ^{13}O ± SD***	
**Early Eocene: Vastan Lignite Mine (India) & Ghazij (Pakistan) Fauna**						
**Perissodactyla**						
**Cambaytheriidae**						
Cambaytherium sp.	Terrestrial	7	-10.8	±0.9	25.3	±0.7
**Artiodactyla**						
**Dichobunidae**						
Gujaratia indica	Terrestrial	1	-10.9		24.8	
**Condylarthra**						
**Quettacyonidae**						
Sororocyon sp.	Terrestrial	7	-10.4	±0.9	26.6	±0.7
**Tillodontia**						
Undescribed**	Terrestrial	3	-11.4	±1.0	26.6	±0.7
**Creodonta**						
**Hyaenodontidae**						
Undescribed	Terrestrial	1	-11.5		24.9	
**Middle Eocene: Kuldana and Gali Jhagir Fauna (Pakistan)**						
**Perissodactyla**						
**Anthracobunidae**						
Anthracobune pinfoldi	Terrestrial	3	-9.0	±0.8	23.5	±1.2
Anthracobune wardi	Terrestrial	4	-9.6	±0.5	25.7	±1.8
Obergfellia occidentalis	Terrestrial	1	-8.9		24.3	
Unknown	Terrestrial	1	-9.3		26.8	
**Rhinocerotidae**						
Jhagirolophus	Terrestrial	1	-8.7		26.8	
**Artiodactyla**						
**Raoellidae**						
Indohyus sp.	Semi-aquatic	4	-10.1	±1.2	21.0	±0.4
Khirtharia dayi	Terrestrial	7	-8.9	±1.0	24.1	±1.1
**Unknown**						
Undescribed	Terrestrial	$2^{\#}$	-6.8	±0.1	27.0	±1.0
**Cetacea**						
**Pakicetidae**						
Undescribed	Semi-aquatic	1	-8.8		21.5	
Ichthyolestes pinfoldi	Semi-aquatic	2	-12.6	±0.4	21.9	±0.3
Nalacetus sp.	Semi-aquatic	1	-13.3		25.3	
Nalacetus ratimitus	Semi-aquatic	1	-11.7		24.1	
Pakicetus attocki	Semi-aquatic	3	-13.2	±1.5	23.6	±2.6
Pakicetus calcis	Semi-aquatic	1	-13.3		20.0	
Pakicetus chittas	Semi-aquatic	1	-9.0		22.0	

**Ambulocetidae**						
Ambulocetus natans	Semi-aquatic	2	-14.2	±0.6	22.9	±0.4
**Remingtonocetidae**						
Attockicetus praecursor	Semi-aquatic	1	-11.3		22.2	
Attockicetus praecursor	Semi-aquatic	1	-6.5		24.3	
**Middle Eocene: Kalakot Fauna (India)**						
**Perissodactyla**						
**Anthracobunidae**						
Anthracobune wardi	Terrestrial/ Semi-aquatic	2	-9.0	±0.3	21.5	±0.3
**Rhinocerotidae**						
Hyrachyus asiaticus	Terrestrial	2	-9.1	±0.1	20.9	±0.3
Undescribed rhinocerotoid	Terrestrial	1	-9.3		24.7	
**Hyracodontidae**						
Undescribed	Terrestrial	2	-8.9	±0.2	24.4	±1.8
**Tapiroidea**						
Kalakotia	Terrestrial	6	-9.3	±0.5	22.6	±1.3
**Artiodactyla**						
**Raoellidae**						
Indohyus sp.	Semi-aquatic	3	-8.7	±1.9	23.4	±1.4
Bunodentus sp.	Semi-aquatic	5	-8.6	±0.2	22.7	±1.8

*When only 2 specimens were sampled, the range in values relative to the mean is reported in place of the standard deviation

#Mean value reported for an upper and lower third molar sampled from the same specimen (H-GSP 97064)

** Values are reported from Table 1 of Clementz et al [119] and are the correct values. Some identifications in their Appendix were reversed.
