# Supplementary material for: Anthracobunids from the Middle Eocene of India and Pakistan Are Stem Perissodactyls
Source: PLoS One. 2014 Oct 8;9(10):e109232. doi: 10.1371/journal.pone.0109232 (PMC4189980; doi:10.1371/journal.pone.0109232)
Supplement: Table S9 — Taxonomy, specimen number, tooth identification, and stable isotope values of fossil enamel samples used in this study. (PDF) [file pone.0109232.s014.pdf]

Taxon	Age	Region of Provenance	Specimen Number	Sample	$\delta^{13}C$ ‰	$\delta^{18}O$‰
**Artiodactyla**						
**Raoellidae**						
Indohyus sp.	Middle Eocene	Kalakot	RR 61	M^x	-11.4	21.4
Indohyus sp.	Middle Eocene	Kalakot	RR 93	M_x	-10.5	20.7
Indohyus sp.	Middle Eocene	Kalakot	RR 94	M^x	-9.7	20.6
Indohyus sp.	Middle Eocene	Kalakot	RR 97	M^x	-8.7	21.2
Indohyus sp.	Middle Eocene	Kalakot	RR 607	M1-2/	-8	24.0
Indohyus sp.	Middle Eocene	Kalakot	RR 608	M/3	-10.8	24.4
Indohyus sp.	Middle Eocene	Kalakot	RR 609	M/2	-7.3	21.8
Bunodentus sp.	Middle Eocene	Kalakot	RR 610	M/1	-8.7	21.2
Bunodentus sp.	Middle Eocene	Kalakot	RR 611	M/2-3	-8.8	24.5
Bunodentus sp.	Middle Eocene	Kalakot	RR 612	M/2	-8.5	23.7
Bunodentus sp.	Middle Eocene	Kalakot	RR 613	M2/	-8.3	20.2
Bunodentus sp.	Middle Eocene	Kalakot	RR 614	M/3	-8.7	23.7
Khirtharia dayi	Middle Eocene	Ganda Kas (Loc. 62)	H-GSP 96360	M/3	-8.9	23.0
Khirtharia dayi	Middle Eocene	Ganda Kas (Loc. 62)	H-GSP 92023	M2/	-10.6	23.8
Khirtharia dayi	Middle Eocene	Ganda Kas (Loc. 62)	H-GSP 18397	M2/	-8.7	25.3
Khirtharia dayi	Middle Eocene	Ganda Kas (Loc. 62)	H-GSP 30228	M/3	-9.5	23.7
Khirtharia dayi	Middle Eocene	Kuldana	GSP-UM 694	n/a	-8.2	24.7
Khirtharia dayi	Middle Eocene	Kuldana	?	n/a	-7.6	22.5
Khirtharia dayi	Middle Eocene	Kuldana	?	n/a	-8.9	25.4
**Dichobunidae**						
Gujaratia indica	Early Eocene	Vastan Lignite Mine	IITR-SB-VLM 887	M^3	-10.9	24.8
Undescribed artiodactyl	Middle Eocene	Shepherd's Lake (H-GSP 9607)	H-GSP 97064	M^2	-6.9	25.9
Undescribed artiodactyl	Middle Eocene	Shepherd's Lake (H-GSP 9607)	H-GSP 97064	M^3	-6.8	27.0
**Cetacea**						
**Pakicetidae**						
Pakicetid?	Middle Eocene	Shepherd's Lake (H-GSP 9607)	H-GSP 96631	Incisor	-8.8	21.5
Ichthyolestes pinfoldi	Middle Eocene	Ganda Kas (Loc. 62)	H-GSP 96553	dP3/	-12.2	22.2
Ichthyolestes pinfoldi	Middle Eocene	Ganda Kas (Loc. 62)	H-GSP 91047	dP/3	-13	21.6

Nalacetus sp.	Middle Eocene	Ganda Kas (Loc. 62)	H-GSP 96055	P4/	-13.3	25.3
Nalacetus ratimitus	Middle Eocene	Ganda Kas (Loc. 62)	H-GSP 91036	C/1	-11.7	24.1
Pakicetus attocki	Middle Eocene	Ganda Kas (Loc. 62)	H-GSP 18470	P4/	-14.7	21.5
Pakicetus attocki	Middle Eocene	Ganda Kas (Loc. 62)	H-GSP 18519	C1/	-13.1	26.5
Pakicetus attocki	Middle Eocene	Ganda Kas (Loc. 62)	H-GSP 91034	dP2/	-11.7	22.8
Pakicetus calcis	Middle Eocene	Shepherd's Lake (H-GSP 9607)	H-GSP 96505	P/4	-13.3	20
Pakicetus chittas	Middle Eocene	Shepherd's Lake (H-GSP 9607)	H-GSP 97254	P4/	-9	22
**Ambulocetidae**						
Ambulocetus natans	Middle Eocene	Ganda Kas (Loc. 9209)	H-GSP 18507		-14.1	22.7
Ambulocetus natans	Middle Eocene	Ganda Kas (Loc. 9209)	H-GSP 18507		-14.1	23.3
Ambulocetus natans	Middle Eocene	Ganda Kas (Loc. 9209)	H-GSP 18507		-12.5	23.9
Ambulocetus natans	Middle Eocene	Ganda Kas (Loc. 9205)	H-GSP 96129	P/1	-14.8	22.5
**Remingtonocetidae**						
Attockicetus praecursor		Shepherd's Lake (Loc. 9607)	H-GSP 96630	P3/	-11.3	22.2
Attockicetus praecursor		Ganda Kas (Loc. 9204)	H-GSP 96232	P4/	-6.5	24.3
**Perissodactyla**						
**Cambaytheriidae**						
Cambaytherium	Early Eocene	Vastan Lignite Mine	IITR-SB-VLM-939	P^4	-10.3	25.1
Cambaytherium	Early Eocene	Vastan Lignite Mine	IITR-SB-VLM-939	P^4	-9.8	24.5
Cambaytherium	Early Eocene	Vastan Lignite Mine	IITR-SB-VLM-545	P_4	-9.8	25.3
Cambaytherium	Early Eocene	Vastan Lignite Mine	IITR-SB-VLM-502	M^3	-10.7	24
Cambaytherium	Early Eocene	Vastan Lignite Mine	IITR-SB-VLM-795	M_3	-11.5	25.9
Cambaytherium	Early Eocene	Vastan Lignite Mine	IITR-SB-VLM-811	P_4	-10.1	26.3
Cambaytherium	Early Eocene	Vastan Lignite Mine	IITR-SB-VLM-7761	M_3	-11.7	25.4
Cambaytherium	Early Eocene	Vastan Lignite Mine	IITR-SB-VLM-7726	M_3	-12.1	25.2
**Anthracobunidae**						
Anthracobune pinfoldi	Middle Eocene	Ganda Kas	H-GSP 92030	M_x	-10.75	24.8
Anthracobune pinfoldi	Middle Eocene	Ganda Kas	H-GSP 92030	M_x	-9.8	22.8

Anthracobune pinfoldi	Middle Eocene	Ganda Kas	H-GSP 92030	M_x	-9.1	25.3
Anthracobune pinfoldi	Middle Eocene	Ganda Kas	H-GSP 97106	M_3	-8.9	24.2
Anthracobune pinfoldi	Middle Eocene	Ganda Kas	H-GSP 82-31P	M_3	-8.2	22.1
Anthracobune wardi	Middle Eocene	Ganda Kas	H-GSP 30229	P^2	-8.9	27.3
Anthracobune	Middle Eocene	Ganda Kas	H-GSP 96214	M_3	-9.3	26.8
Anthracobune wardi	Middle Eocene	Ganda Kas	H-GSP 96434	M_3	-10.1	24.1
Anthracobune wardi	Middle Eocene	Ganda Kas	H-GSP 96052	M_3	-9.5	24.3
Anthracobune wardi	Middle Eocene	Ganda Kas	H-GSP 96258	M_3	-9.9	27.2
Anthracobune wardi	Middle Eocene	Kalakot	RR 361	P/3	-9.3	21.8
Anthracobune wardi	Middle Eocene	Kalakot	RR 411	M^3	-8.79	21.29
Obergfellia occidentalis	Middle Eocene	Ganda Kas (Loc. 9606)	H-GSP 96149	?	-8.9	24.3
**Hyracodontidae**						
Undescribed	Middle Eocene	Kalakot	RR 603	?	-9.0	22.6
Undescribed	Middle Eocene	Kalakot	RR 359 (MC 8)	M/x	-8.7	26.1
**Rhinocerotoidea**						
Hyrachyus asiaticus	Middle Eocene	Kalakot	ONGC/K/21	M3/	-9.1	21.1
Hyrachyus asiaticus	Middle Eocene	Kalakot	ONGC/K/21	M3/	-9.1	20.6
Jhagirolophus	Middle Eocene	Gali Jhagir (Loc. 9709)	H-GSP 97142	Mx/	-8.7	26.8
Rhinocerotoid	Middle Eocene	Kalakot	RR 604	M/2	-9.3	24.7
**Tapiroidea**						
Kalakotia	Middle Eocene	Kalakot	RR 353	M3/	-9.9	20.8
Kalakotia	Middle Eocene	Kalakot	RR 353	M/3	-9.5	21.5
Kalakotia	Middle Eocene	Kalakot	RR 355	M3/	-8.9	22.4
Kalakotia	Middle Eocene	Kalakot	RR 356	M3/	-9	24.1
Kalakotia	Middle Eocene	Kalakot	RR 357	M3/	-8.8	23.2
Kalakotia	Middle Eocene	Kalakot	RR 364	M3/	-9.8	23.5
**Creodonta**						
Hyaenodontidae						

Undescribed	Early Eocene	Vastan Lignite Mine	IITR-SB-VLM-782	M3	-11.5	24.9
**Condylarthra**						
**Quettacyonidae**						
Sororocyon sp.	Eocene (Ypresian)	Ghazij Formation	GSP-UM 4144	n/a	-10.1	26.6
	Eocene (Ypresian)	Ghazij Formation	GSP-UM 4144	n/a	-9.1	26.3
	Eocene (Ypresian)	Ghazij Formation	GSP-UM 4144	n/a	-11.1	25.7
	Eocene (Ypresian)	Ghazij Formation	GSP-UM 4133	n/a	-9.6	27
	Eocene (Ypresian)	Ghazij Formation	GSP-UM 4132	n/a	-11.7	26.4
	Eocene (Ypresian)	Ghazij Formation	GSP-UM 4144	n/a	-10.9	28
	Eocene (Ypresian)	Ghazij Formation	GSP-UM 4172	n/a	-10.4	26.2
**Tillodontidae**						
Undescribed	Eocene (Ypresian)	Ghazij Formation	GSP-UM 4147	n/a	-12.4	25.8
	Eocene (Ypresian)	Ghazij Formation	GSP-UM 4131	n/a	-11.1	27.1
	Eocene (Ypresian)	Ghazij Formation	GSP-UM 4155	n/a	-10.6	27.1
**Mesonychidae**						
Mesonychid ident.	Middle Eocene	Kalakot	ONGC/K/19	Mx	-9.4	22.1
