# Supplementary material for: Anthracobunids from the Middle Eocene of India and Pakistan Are Stem Perissodactyls
Source: PLoS One. 2014 Oct 8;9(10):e109232. doi: 10.1371/journal.pone.0109232 (PMC4189980; doi:10.1371/journal.pone.0109232)
Supplement: Text S1 — Additional taxonomic description. (DOCX) [file pone.0109232.s017.docx]

**Additional Taxonomic Description**

***Anthracobune* Pilgrim, 1940**

# *Anthracobune pinfoldi* Pilgrim, 1940

***Referred specimens****.* The following specimens were investigated as original or cast:

BMNH M. 15793 (left m3; ‘Lammidhan’, Ganda Kas Area, Punjab Province, Pakistan);

BMNH M. 15795 (partial maxilla with left P2-3, roots of P1, and C alveolus; holotype of *A. (?) daviesi*, ‘Lammidhan’);

BMNH 32169 (right m1; ‘Lammidhan’);

H-GSP 82-31P (left P3-M3 and right scapula fragment; H-GSP Locality 146, Ganda Kas Area);

H-GSP 92030 (mandible with m3 fragment; H-GSP Locality 9203, Ganda Kas Area);

H-GSP 92124 (mandible with m3 roots; H-GSP Locality 9206, Ganda Kas Area);

H-GSP 97106 (left p1, p4, m1, m2; right m1; left P4-M3; right I3, base of C, P1, P2, P4-M3, skull including rostrum and poorly preserved petrosals, forelimb bones including carpals, and phalanges, femur, cranial, and postcranial material; H-GSP Locality 9611, Thatta North Area, Punjab Province).

# *Anthracobune wardi* Dehm and Oettingen-Spielberg, 1958

***Referred*** *s****pecimens*.** The following specimens were studied as originals or casts:

GSP-UM 103 (right m3, Chorlakki, North-West Frontier Province, Pakistan);

GSP-UM 474 (left p2, Chorlakki);

GSP-UM 519 (trigonid, Chorlakki);

GSP-UM 1549 (right p4, Chorlakki, sometimes referred to as 549);

GSP-UM 615 (left M2, Chorlakki);

GSP-UM 687 (right M3, Chorlakki);

GSP-UM 1519 (left mx trigonid, Chorlakki);

H-GSP 538 (left M3, Ganda Kas Area);

H-GSP 982 (dp3-4, Ganda Kas Area);

H-GSP 1633 (left m3, Ganda Kas Area);

H-GSP 1975 (right M3, Ganda Kas Area);

H-GSP 92032 (hypocone, H-GSP Locality 66, Ganda Kas Area);

H-GSP 96052 (right dp3-4, p4 in crypt, m1, left dp2-4, m1 fragment, H-GSP Locality 62);

H-GSP 96258 (complete left and right mandible with right and left c-m3, H-GSP Locality 62);

H-GSP 96434 (complete left and right mandible with left and right p1-m3, H-GSP Locality 62);

H-GSP 30229 (left P2, H-GSP Locality 62);

H-GSP 30349 (juvenile jaw with crowns for dp2-4; H-GSP Locality 62, published erroneously as 30489 by Thewissen et al., 2007);

IPHG 1956 II 12 (left Mx, Ganda Kas);

IPHG 1956 II 14 (right M3, Ganda Kas Locality 24 of Dehm and Oettingen-Spielberg, 1958);

IPHG 1956 II 16 (left p2, identified as p4 in its description, Ganda Kas Locality 10 of Dehm and Oettingen-Spielberg, 1958);

IPHG 1956 II 20 (right Mx, holotype of *Pilgrimella pilgrimi* Dehm and Oettingen-Spielberg, 1958, Ganda Kas Locality 24 of type description);

IPHG 1956II 21 (left M2; Ganda Kas Locality 1 of Dehm and Oettingen-Spielberg, 1958);

IPHG 1956II 22 (left Mx fragment, Ganda Kas Locality 24 of Dehm and Oettingen-Spielberg, 1958);

IPHG 1956II 23 (Mx fragment Ganda Kas Locality 22 of Dehm and Oettingen-Spielberg, 1958);

LUVP 15006 (right P2-M2, Lucknow University Locality 1005, Kalakot Area, Jammu & Kashmir, India);

VPL/K/544 (left p4, Kalakot);

RR 361 (right p2, Kalakot);

RR 411 (skull fragment with M1-3, Kalakot);

WIF/A 1101 (left p1-m3 and right i2-3, p2-m3, holotype of *Anthracobune aijiensis*, Subathu Formation, Kalakot);

WIF/A 616 (left P2-4, Kalakot).

***Obergfellia*, new genus.**

**Referred specimens**. The following specimens were studied as originals or casts:

H-GSP 538, left M2 (Ganda Kas Area);

H-GSP 568, right M3 (Ganda Kas Area)

H-GSP 96214 (right m3 in posterior part of mandible with angular process)

H-GSP 96544 (right P2, H-GSP Locality 62, Ganda Kas Area);

H-GSP 96149 (mandible with alveoli for i1-m3 on both sides, and worn crowns for right p3-m3 and left m1-3, H-GSP locality 9606, Ganda Kas Area);
